# Online videos indicate human and dog behaviour preceding dog bites and the context in which bites occur

**DOI:** 10.1038/s41598-018-25671-7

**Published:** 2018-05-08

**Authors:** Sara C. Owczarczak-Garstecka, Francine Watkins, Rob Christley, Carri Westgarth

**Affiliations:** 1 Department of Epidemiology and Population Health, Institute of Infection and Global Health, University of Liverpool, Liverpool, L69 7BE UK; 2 Institute for Risk and Uncertainty, University of Liverpool, Liverpool, L69 7ZF UK; 3 Department of Public Health and Policy, Institute of Psychology, Health and Society, University of Liverpool, Liverpool, L69 3GL UK

## Abstract

YouTube videos of dog bites present an unexplored opportunity to observe dog bites directly. We recorded the context of bites, bite severity, victim and dog characteristics for 143 videos and for 56 videos we coded human and dog behaviour before the bite. Perceived bite severity was derived from visual aspects of the bite. Associations between bite severity and victim, dog and context characteristics were analysed using a Bayesian hierarchical regression model. Human and dog behaviour before the bite were summarised with descriptive statistics. No significant differences in bite severity were observed between contexts. Only age of the victim was predictive of bite severity: adults were bitten more severely than infants and infants more severely than children. Non-neutral codes describing dog body posture and some displacement and appeasement behaviours increased approximately 20 seconds before the bite and humans made more tactile contacts with dogs 21 seconds before the bite. This analysis can help to improve understanding of context in which bites occur and improve bite prevention by highlighting observable human and dog behaviours occurring before the bite.

## Introduction

Dog bites are a global public health problem resulting in substantial costs to health care systems^1–3^, and businesses as a result of time off work, human physical and mental health impacts^4–7^ and also affect dog welfare, since dogs that bite are likely to be relinquished to shelters^8^ and/or euthanised^9^. Human population-level risk factors associated with dog bites include young age of the victim^1,10–14^ (but see^15,16^) and male sex^11^ (but see^12,15,16^). The breed, neuter status and sex of dogs have also been highlighted^17^, although the link between these factors and bite risk are contested^11,15^. Additionally, the physical environment where the interactions are taking place and the dog’s history are suggested risk factors for the occurrence of a bite^5,18^. Most bites to adults are to limbs and children receive more bites to the face and neck areas^1^, regardless of dog size^19^, suggesting that children interact with dogs differently than adults.

As well the risk factors for the occurrence of a bite, studies have scrutinised the risk factors for severity of a bite. The severity of a bite tends to be greater among older victims, when the victim is not the owner of the biting dog, when the bite takes place in a public area and outside of the play context^15^. A link between severity and breed has also been suggested^20,21^ (but see^22^), however lack of clear guidelines for breed identification and small sample sizes makes this finding unreliable and inconclusive^23^. Improving understanding of what changes the severity of bites is important, as whilst some bites may be difficult to prevent, reducing their severity may be more achievable.

Understanding of the contexts in which dog bites occur is crucial for bite prevention. Interactions that are often discussed as preceding bites at a population level include those that are likely to be painful or uncomfortable to dogs, such as medical procedures, physical abuse to dogs^16,18^, teasing^10^, interacting with dogs over resources (e.g. food or toys) or on a dog’s perceived territory^24^, playing with or nearby a dog^25^ and mundane, daily occurrences such as petting or reaching towards a dog^13,24,26,27^. However, a qualitative study illustrated that some bite victims could not explain why they were bitten or were not aware of the dog’s presence before the bite^26^, which suggests that identification of interactions before the bite may not be very accurate.

Dog bites cannot be studied experimentally as exposing a volunteer to a bite or provoking a dog to bite would be unethical. As bite incidents are relatively rare, collecting data through real-time observations is not feasible. Therefore, dog bite data is gathered through general population surveys e.g.^28^, veterinary caseloads e.g.^17^, hospital admissions e.g.^1^ and interviews with dog bite victims e.g.^26^. The hospital admission datasets are often large, but the data does not systematically include information about the circumstances of the bite^29^. Some of the data, e.g. regarding dog’s breed, may also be unreliable or not recorded^23,30^. Moreover, in UK hospitals, dog bites are coded under the code “Bitten or struck by dog”, which means that other dog-related incidents, such as falling over because of a dog, may be included within these statistics^1^. As only a fraction of bites warrant a visit to a hospital^31–33^, hospital derived-data does not represent all types of bites and bites that do not warrant medical attention have been under studied^32^. Data collected by reviewing veterinary referral cases is also biased to those who are willing to pay for behavioural referral and it is plausible that this data over-represents large dogs as owners tolerate aggression in smaller dogs for longer^34^. Surveys and questionnaires regarding being bitten often rely on convenience sampling, which may lead to a self-selection bias. Detailed interviews with dog bite victims or witnesses of dog bites are an alternative to the above methods^26,35^, however the sample size is typically small.

Video sharing platforms, such as YouTube, offer an opportunity to address some of the above issues. YouTube has been used to study sequential behaviours and human-dog interactions within the context in which they occur (e.g. during dog-training) and to collect a more diverse sample of behaviours than veterinary caseload data permits^36,37^. YouTube provides a chance to observe the interactions leading to a bite directly, in a naturalistic context. This is important as bite education strategies are often structured around the ladder of aggression^38^. This theory proposes that dog behaviours before a bite escalate gradually (in the time immediately before the bite or over the years), with some behaviours (like lip licking or head turning), being shown earlier in time than other behaviours (like growling or teeth-barring^38^).

This study has the following aims: 1) to summarise the contexts in which dog bites occur and to describe victim and dog characteristics using YouTube videos of bites, 2) to describe human and dog behaviour preceding a bite, 3) to examine factors that predict the perceived severity of a bite using variables extracted from YouTube videos, and 4) to evaluate YouTube as a novel method of collecting data about dog bites.

## Methods

### Video sampling

Between January 2016 and March 2017, YouTube videos were searched using the following terms: “dog bite”, “dog attack”, “dog bites man/woman”, “dog bites child” and “kid gets bitten”. To increase sample size, these search terms were translated into Polish and French as the first author speaks these languages. Dog bite was defined as a dog holding a person’s body part in their mouth and applying pressure, which could be reflected by a bite mark and/or the victim’s vocalisations (e.g. screaming) or facial expressions indicative of pain (e.g. grimacing). We excluded videos under 5 seconds, that showed bite compilations, ‘bite work’ (i.e. any activity involving teaching a dog to bite or biting on command), or where a bite was not visible (due to quality or content of a video). All identified videos (N = 653) were watched by the first author and, after excluding duplicates, 143 videos were included in the final sample. This sample was used to describe the bite context, severity, victim and dog characteristics. Fifty-six videos from this sample showed the behaviour of a dog and a person in detail from the beginning of an interaction until a bite and were included in analysis of pre-bite behaviour.

### Video description

Details of victim, dog, context and bite characteristics were extracted from each video (Table 1). Bite severity is usually approximated by asking if a bite required medical attention or by inspecting the wound^39,40^. As this was not possible for all videos, we developed a scale, which incorporated different elements of bites more easily discerned from the videos and expressed ‘perceived severity’. When constructing this measure, the importance of puncture wounds was emphasised, because bites that result in a puncture have been the basis of previous bite severity scales^39,40^. We assumed that the puncture did not occur when it was not possible to ascertain whether a bite broke the skin. Dog head shaking whilst biting was highlighted as it can lead to further lacerations of existing wounds^40^. The duration of the bite was included as bites that are longer could be more traumatic. A cut-off point for bite duration was set at one second because most bites observed here were less than that. Where a video showed multiple bites of different severity, the most extreme scores for variables a, b and c were included to calculate the total score. Perceived severity is defined as (1):

1 $$perceived\,severity={\rm{n}}\,\ast \,{\rm{a}}+{\rm{b}}+{\rm{c}}$$

**Table 1:** Variables describing dog, victim, context and bite characteristics.

Variable	Categories
Dog size	Small- dogs that appear shorter than 30 cm at withers Medium- dogs between 30 and 45 cm at withers Big- dogs that appear above 45 cm at withers
Dog breed	A range of breeds were identified. When it was impossible to reasonably determine a breed, a dog was classed as a crossbreed. Breeds were identified with reference to the UK Kennel Club Breed Information Centre^41^
Victim’s age	Infant– based on visual characteristics, a young child that does not yet appear to be fully stable when walking, a child that is crawling or using elements of the environment when walking or standing up Child/teenager– a child that is fully comfortable walking but does not appear to be fully mature Adult– a mature person
Victim’s sex	Male Female
Site of the bite	Limbs Head or face Other– e.g. torso Multiple
Severity of the bite	Total score
Initiator of interaction	Dog Victim NA- not possible to discern
Handler’s sex	Male Female NA– not visible or not present
Location	Indoor Outdoor

where:

n = number of bites to the victim observed in the video

$${\rm{a}}=\{\begin{array}{l}{\rm{3}}\,\mbox{--}\,{\rm{puncture}}\,{\rm{wound}}\,{\rm{is}}\,\mathrm{visible}\,\\ {\rm{1}}\,\mbox{--}\,{\rm{puncture}}\,{\rm{wound}}\,{\rm{is}}\,{\rm{not}}\,{\rm{present}}\,{\rm{or}}\,{\rm{not}}\,\mathrm{visible}\,\end{array}$$

$${\rm{b}}=\{\begin{array}{l}{\rm{1}}\,\mbox{--}\,{\rm{dog}}\,{\rm{shook}}\,{\rm{its}}\,{\rm{head}}\,{\rm{whilst}}\,\mathrm{biting}\,\\ {\rm{0}}\,\mbox{--}\,{\rm{dog}}\,{\rm{did}}\,{\rm{not}}\,{\rm{shake}}\,{\rm{its}}\,{\rm{head}}\,{\rm{whilst}}\,\mathrm{biting}\,\end{array}$$

$${\rm{c}}=\{\begin{array}{c}{\rm{1}}\,\mbox{--}\,{\rm{dog}}\,{\rm{held}}\,{\rm{on}}\,{\rm{for}} > \mathrm{1s}\,\\ {\rm{0}}\,\mbox{--}\,{\rm{dog}}\,{\rm{held}}\,{\rm{on}}\,{\rm{for}}\le \mathrm{1s}\,\end{array}$$

To describe the interaction preceding the bite without over-relying on inferring the dog’s motivation or emotions, we adapted a classification used by Reisner *et al*.^24^ (Table 2) and each bite was assigned to one context.

**Table 2:** Interactions preceding the bite adapted from Reisner *et al*.^24^.

Context labels	Definition
Resources	Manipulating with dog’s food, treats, bones, or toys
Benign	Petting, kissing, bending over or reaching above, hugging, reaching towards, speaking to or walking by or with a dog
Resting	Interacting with a dog whilst the dog is resting, including removing from furniture and laying besides a dog
Unpleasant	Shouting, pulling on a collar, restraining, grooming, drying with a towel, lifting, nail clipping, throwing an object at a dog and missing
Painful	Hitting, stepping or falling onto a dog, throwing and object at a dog without a miss, pulling hair, jerking on the lead/collar
Territorial	Bites that occur within what a dog may perceive as their territory (e.g. a yard, by the fence, garden etc.)
Public space	Bites that occur outdoors, in an area that is unlikely to be dog’s territory (e.g. in the park or on the street)
Play	Interactions when dog is showing a playful body language, i.e. loose body, play bows, jerky and exaggerated movement.

### Ethogram development and behaviour coding

Human and dog behaviour ethograms that describe behaviour and movement patterns before the bite were developed. The dog behaviour ethogram (Supplement 1) was constructed to include behaviours described as preceding a dog bite and often taught as a part of a dog bite prevention initiatives^38^, which include displacement activities (such as a shake off, yawning) and appeasement gestures (like head turning or nose licking). In addition, the following behaviours were included: locomotory behaviours (direction in relation to the person and pace), body, tail and ear posture (as these are associated with negative affect in dogs^42^), body position, vocalisation and the type of contact that a dog made with a person (gentle or intensive).

To describe human behaviour preceding bites, the following behaviours were included: macro-movements near the dog (i.e. head and body turns, standing over a dog, moving legs, arms or objects towards the dog), types of tactile contact with a dog, (i.e. petting, hugging, hitting, restraining, pushing, pulling, holding a body part and lifting), vocalisations, body position and locomotory behaviours (direction in relation to the dog and pace). The descriptions of human behaviours are based on previous studies exploring human-dog play interactions^43^ (see Supplement 2 for human behaviour ethogram).

We also noted the site of contact on the body and body part used during contact for both person and a dog. Dogs were coded to make a tactile contact with a person using: head/neck area, mouth, limbs (including tail) and body and a person was coded to make a contact with a dog using head/neck area, limbs or body. Both dogs and people were coded as being touched on the head/neck area, limbs, or body.

The definitions of the behaviours/ behaviour units included in the final ethograms were tested using a sample of the final video dataset, in order to refine the definitions if needed, and to ensure that the behaviours selected were described in a narrowest possible way, but remained broad enough to be able to identify them across different styles of videos. The videos were coded from beginning of each clip or a beginning of a human-dog interaction (if a dog and person were not both in the video at the beginning) until the first bite. The ethograms were applied via scan sampling. For this, the videos were observed for each 3 second interval from the beginning of the video/interaction until the bite and all the behaviours listed in the ethograms which occurred within each 3 second window were noted.

### Observer reliability

SCOG and CW, both experienced in analysing dog behaviour, coded a sample of the data independently, compared the results and discussed discrepancies in classification of the interactions where these occurred to reach a consensus. Subsequently, all videos were coded twice by SCOG (in January and March 2016) and intra-rater agreement was calculated using Cohen’s kappa. A number of more subjective variables described in Table 1 (dog size, victims’ age, dog breed and bite severity score) were checked for inter-observer reliability with an observer naïve to the purpose of the study and dog behaviour literature using 10% of randomly selected dog bite videos and the Cohen’s Kappa was calculated. For both intra- and inter-rater reliability a threshold of 0.61–0.80 was considered acceptable.

### Statistical analysis

#### Behaviours preceding a bite

All statistical analyses were conducted using R^44^. To summarize the behavior before the bite, videos across all contexts were pooled and a percentage of occurrence within a given time frame before the bite was provided. We limited the analysis to 35 seconds before the bite as only 5 videos were longer than this.

#### Bite severity

To understand the association between bite severity score and context, victim and dog characteristics, we used a hierarchical regression model. The distribution of the bite severity scores was checked and data were assumed gamma distributed, as on visual inspection the data fit the gamma model better than models for positive integers, e.g. Poisson. Bite severity scores were the dependent variable in these models and were modelled (using a log-link) as a function of: bite context, the duration of the interaction in seconds, dog size, victim sex, victim age, the anatomical location of the bite, and whether the human or dog initiated the interaction. The model was hierarchical because varying intercept parameters were included for different bite contexts, and those intercepts were constrained by a common distribution. This approach reflected that the bite contexts are not completely independent of one another but are a subset of possible categorisations. This allowed partial-pooling of bite severity estimates across contexts, which often results in more accurate predictions^45^, particularly when the number of data points per hierarchical group (e.g. the context of a bite) are highly variable^46^, which was the case here. For comparison, we also display the sample mean bite severity and 95% confidence interval (CI), derived from non-parametric bootstrapping, for each bite context.

To account for uncertainty in model predictions, the analyses were computed using a Bayesian approach^45^ utilising Markov chain Monte Carlo (MCMC) with the probabilistic programming language JAGS version 4.2^45^ through the *runjags* package in R^44^. We used model selection to assess whether all of the predictor variables were necessary for predicting bite severity. The baseline model included the bite contexts, the duration of the interaction and dog size, since these variables were considered *a priori* important for predicting bite severity. Thirteen additional models were computed including all combinations of the remaining predictor variables noted above. The best fitting model was recomputed with bite contexts as a fixed effect rather than a varying effect, to assess whether a hierarchical model was necessary. Models were assessed using the widely applicable information criterion (WAIC), a Bayesian information criterion that evaluates the out-of-sample predictive accuracy of a model relative to other possible models. Information criteria are preferable to classical measures of model fit (e.g. *R*^2^) because they guard against under- and over-fitting to the data^46^. The WAIC values were transformed to WAIC weights, giving the relative probability of each model having the best out-of-sample predictive accuracy. Fourteen videos (around 10%) had missing data for who initiated the interaction, so models that included the initiated predictor imputed missing values (see Supplement 3 and Supplement Table 1 for more details).

Prior distributions on regression parameters were broad except for predictor variable coefficients, which had normally distributed priors with means of 0 and standard deviations of 1, further guarding against spurious results in addition to the model selection. The models were run with four MCMC chains, long enough for the effective sample size for each parameter to be >10,000 and Gelman-Rubin statistics to be $<$1.01^46^. Parameters were summarised by their mean and 95% highest density interval (HDI), the 95% most probable parameter values (see Supplement 4 for the R script, and Supplement Table 2 for a full dataset).

### Ethical statement

As all videos were in the public domain, ethical approval from the University Ethics Committee was not required. Videos were used in accordance with YouTube regulations and laws.

## Results

Both intra- and inter- rater agreements on coding were high, and were considered acceptable (κ = 0.91 and κ = 0.73 respectively).

### Video characteristics

Three hundred and sixty-two bites were observed in 143 videos. Most of the observed dogs were cross-breeds (n = 47, 32.87%), other common breeds included: Chihuahuas (n = 13, 9.09%), German Shepherds and Pit bulls (n = 11, 7.69% of each) and Labrador Retrievers (n = 6, 4.2%). Almost half of bites (49.65%) were less than one second long and 74.13% were 3 seconds or less (Supplement Table 2).

The victim, dog, bite and context variables are summarized in Table 3. Male victims were more numerous across all bite contexts and children and infants were more numerous than adults. There were more big dogs compared to medium and small dogs in this sample. The proportion of small dogs that bit in the context of benign interactions was higher than the proportion of medium- and big-sized dogs (43% vs. 17.95% vs. 20.9%). Victims initiated more interactions than dogs (48.95% vs. 41.26%). Bites to limbs were more frequent than bites to any other location. The severity score of most bites did not exceed 5, however 51.67% of all bites that scored over 10 points occurred in the context of territorial interactions (Supplement Table 2).

**Table 3:** Summary of victim, dog, bite and context variables (%).

		Resources	Benign	Resting	Unpleasant	Painful	Territorial	Public Space	Play	TOTAL (n)
Victim sex	Male	4.90	20.59	1.96	5.88	10.78	8.82	11.76	35.29	102
	Female	2.44	36.59	2.44	0	12.20	12.20	17.07	17.07	41
Victim Age	Infant	0	40.74	3.70	7.41	11.11	3.70	18.52	14.81	27
	Child	6.00	22.00	2.00	4.00	6.00	2.00	2.00	56.00	50
	Adult	4.55	21.21	1.51	3.03	15.15	18.18	19.70	16.67	66
Dog Size	Small	8.11	43.24	2.70	5.41	21.62	0	0	18.92	37
	Medium	5.13	17.95	0	2.56	10.26	7.69	20.51	35.90	39
	Big	1.49	20.90	1.49	4.48	5.97	16.42	16.42	32.84	67
Initiator	Dog	1.69	16.95	0	5.08	6.78	18.64	25.42	42	59
	Victim	7.14	38.57	2.86	4.29	17.14	2.86	0	27.14	70
	NA	0	0	0	0	0	7.14	28.57	64.29	14
Location	Indoor	5.80	37.68	1.45	1.45	14.49	1.45	0	37.68	69
	Outdoor	2.70	14.86	1.35	6.76	8.11	17.57	25.68	22.97	74
Site of the bite	Head/Face	6.67	66.67	0	6.67	13.33	0	0	6.67	15
	Limbs	4.67	21.50	1.87	3.74	12.15	9.35	11.21	35.51	107
	Other	0	42.86	0	0	0	0	14.29	42.86	7
	Multiple	0	7.14	0	7.14	7.14	28.57	42.86	7.14	14
Severity	1–5	4.82	33.73	2.41	1.20	15.66	7.23	8.43	26.51	83
	6–10	3.57	17.86	0	10.71	7.14	3.57	10.71	46.43	28
	11–15	5.00	15.00	0	5.00	5.00	10.00	25.00	35.00	20
	16+	0	0	8.33	8.33	0	41.67	33.33	8.33	12
	TOTAL (%)	4.20	25.17	2.10	4.20	11.19	9.79	13.29	30.07	143

### Behaviours preceding a bite

#### Dog behaviour

**Figure 1:**
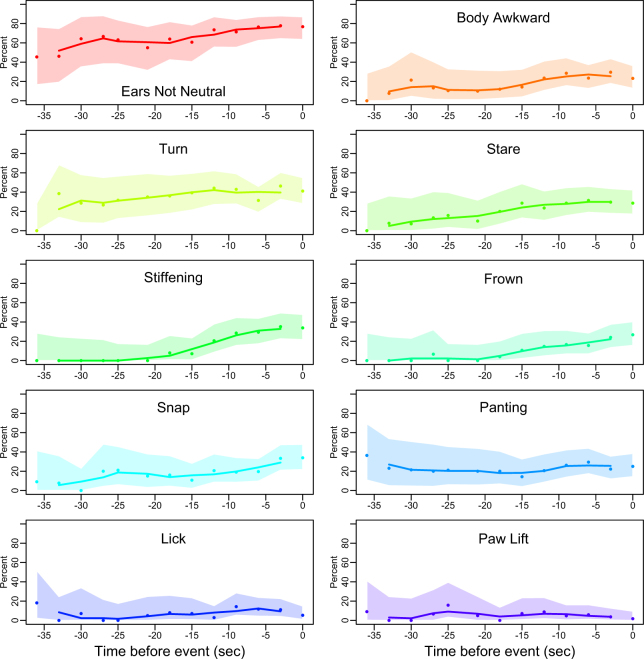
Patterns of changes in dog body carriage (ears and body posture), dog behaviour (head/ body turning, staring, stiffening, frowning, snapping, panting, lip licking, paw lifting) preceding the bite. Dots indicate observed proportions, lines represent 3-point moving averages and the shaded area the 95% confidence intervals for the observations.

The proportion of videos where dogs were seen holding their body awkwardly or in a low position and showing a non-neutral ear carriage increased before the bite. The increase in videos where these changes were observed was seen approximately 30 seconds before the bite for “holding body awkwardly”, 27 seconds before the bite for “body in low position” and 34 seconds before the bite for “non-neutral ear carriage” (Fig. 1). There was no clear pattern of changes in tail carriage and high body posture before a bite.

There was an increase in a proportion of videos where dogs were seen stiffening, snapping and frowning shortly before the bite (from 22, 16 and 22 seconds before the bite respectively). In the lead up to the bite the proportion of videos where dogs were seen head turning, staring, and panting also increase (from 28, 30 and 16 seconds before the bite respectively), but these behaviours decreased, plateaued or fluctuated shortly before the bite.

Yawning and shake off were observed sporadically and lip licking, paw raises and sniffing did not follow any clear pattern (Fig. 1).

There was an increase in the proportion of dogs growling and a decrease in dogs being silent or barking before the bite. Pain-related vocalisations were rare. Closer in time to the bite, more dogs were coded as restrained and fewer were coded as standing. There was no clear pattern regarding play bows, sitting and laying down. As the bite became closer, there was more of fast pace locomotory behaviours and less jumping and slow pace locomotory behaviours. There was no clear pattern regarding dogs making a gentle contact before the bite and there was a clear spike in a proportion of dogs making an intensive contact immediately before the bite, which reflects the moment of a bite. Dogs touched people mainly with their paws and there was no pattern in tactile contact initiated by the dog before a bite (Supplement Table 2).

#### Human behaviour

**Figure 2:**
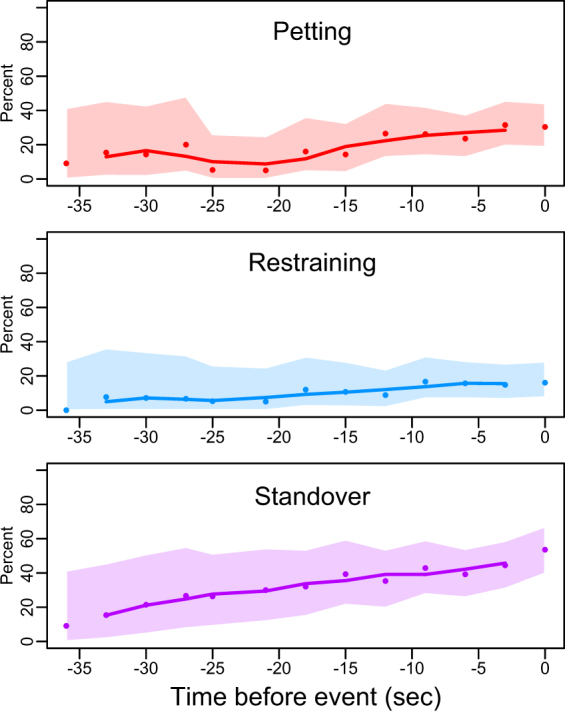
Patterns of changes in human behaviour (petting, restraining and standing over the dog) preceding the bite. Dots indicate observed proportions, clines represent 3-point moving averages and the shaded area the 95% confidence intervals for the observations.

Behaviours grouped as ‘movements without contact with a dog’ were observed more than 50% of the time. Leading to a bite, there was an increase in codes representing standing over a dog (approximately 35 seconds before the bite, Fig. 2). There was no clear pattern to all other non-contact behaviours.

All of the contact behaviours increased approximately 21 seconds before the bite with petting and restraining a dog being particularly frequent (Fig. 2). Hugging, hitting, pushing and pulling did not follow any clear pattern. Kissing, hitting with an object, kicking and pulling hair were not observed or were rare.

Until 21 seconds before the bite there were proportionally more codes for movement towards the dog and from 9 seconds before the bite more codes for movement away from the dog were noted. There was no clear trend regarding changes of pace of movement in time before the bite. There was a sharp increase in the proportion of pain-related vocalisations immediately before the bite (which could indicate anticipation of pain rather than the experience of pain) and a slight increase in laughing vocalisations from 15 seconds before the bite. Normal talk and silence were observed proportionally less often closer in time to the bite. Shortly before the bite standing and crouching behaviours decreased and there were slightly more laying down behaviours (from 18 seconds before the bite). People usually made a tactile contact with a dog using limbs and there was no clear pattern regarding the part of the dog that was touched (Supplement Table 2).

### Model selection

Three models had demonstrably lower WAIC values and higher WAIC weights than the other models assessed (see Supplement 5 for model comparison). The model including varying effects for bite contexts and all predictor variables was ranked first, with WAIC weight of 45%. The same model with bite contexts included as a fixed rather than a varying effect had a WAIC weight of 20%. The model with varying effects for bite contexts, interaction duration, dog size, the anatomical location of the bite and whether the human or dog initiated the interaction and where victim’s sex and age were excluded, had a WAIC weight of 33%. Thus, all predictors appeared important to predicting severity.

#### Bite contexts

**Figure 3:**
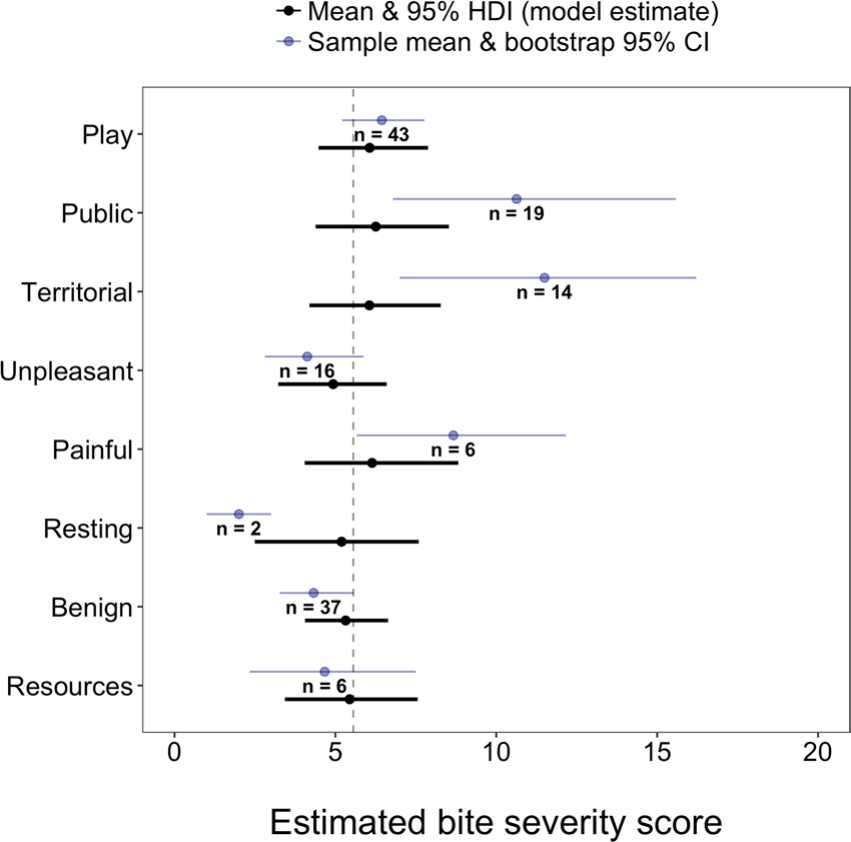
Estimated bite severity in each context. Black points and lines represent the regression model mean and 95% HDI estimates. Blue points and lines represent the raw sample means and bootstrap 95% CIs. Sample sizes are shown next to each parameter. Regression model estimates are pooled towards the overall mean (dashed vertical line) when contexts have relatively low sample size (e.g. resting) and/or deviate greatly from the overall mean without enough data to support such a difference (e.g. territorial).

Across bite contexts, the mean bite severity score was estimated as 5.61 (95% HDI: 4.16, 7.27, see Fig. 2 and Supplement Table 2). Due to the varying numbers of videos in each category, differences among contexts were pooled closer to the overall mean compared to the raw data. The *benign* and *play* contexts have the most influence due to having the largest sample sizes. Moreover, the *territorial* context appeared to have a larger mean estimate but it only had 14 videos with large variation, as evidenced by its 95% CI, resulting in its estimate being pooled towards the overall mean. The intra-class correlation coefficient was both highly uncertain and included zero (mean = 0.11; 95% HDI: 0.00, 0.38), suggesting greater within- than between-context variation for bite severity. The 95% HDI estimates for differences between bite contexts all overlapped zero (see Supplement 6), i.e. we did not observe any significant difference in bite severity between different contexts of interactions (Fig. 3), however, bites in the public space and territorial contexts and painful interactions were more severe than the mean, whereas bites in the context of resting were less severe than the mean, when taking into account just sample mean and bootstrap 95% CI.

#### Other predictor variables

**Figure 4:**
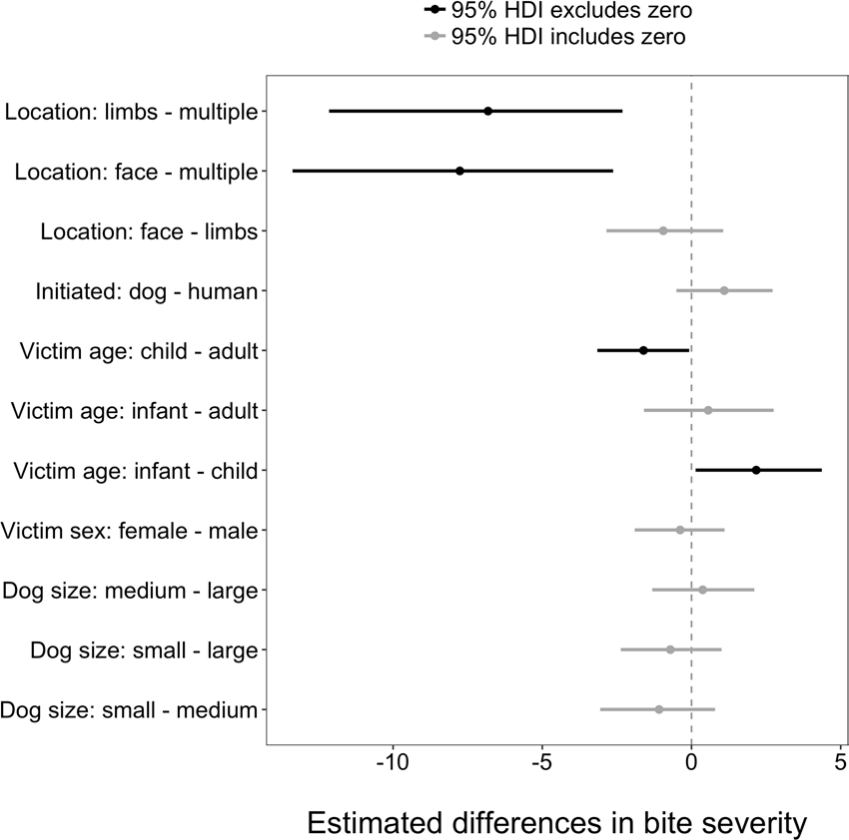
Estimated differences in bite severity between categorical predictor variables. Points and horizontal lines represent mean and 95% HDI model estimates. Estimates in black exclude zero, indicating a significantly non-zero difference; estimates in grey overlap zero.

Among the fixed-effect predictor variables, bite severity scores increased by an average of 1.25 points with approximately every minute (one SD = 56 s) increase in the duration of the interaction (see Supplement 7). The 95% HDI of this estimate extended from 1.09 to 1.41, indicating it was significant. This is somewhat expected as longer videos/longer interactions could feature more bites, which is a variable used for calculating bite severity. Videos with bites to multiple locations had significantly higher bite severity scores than videos with bites only to the limbs (${\beta }_{{limbs}-{multiple}}$ = −6.82; 95% HDI: −12.15, −2.31; Supplement 6) or only to the face (${\beta }_{{face}-{multiple}}$ = −7.76; 95% HDI: −13.37, −2.62), which again could be due to more bites being observed meaning greater severity score. When the victim was an adult, bites were more severe than when the victim was a child (${\beta }_{{child}-{adult}}$ = −1.61; 95% HDI: −3.16, −0.08) and bites to infants were more severe than those to children (${\beta }_{{infant}-{child}}$ = 2.17; 95% HDI: 0.14, 4.37). All other predictor variables had 95% HDI estimates overlapping zero, meaning that they were not significant (Fig. 4; Supplement 6).

## Discussion

In this study, we used YouTube videos to explore dog bites to humans. The most common breeds and types of dogs found in our sample (i.e. German Shepherds, Chihuahuas, Labrador Retrievers and Pit bulls) reflect those previously identified in studies of dog bites^10–13,15–17,47^. Chihuahuas are rarely mentioned in studies that use hospital admissions, possibly because their small size makes them less likely to cause serious injury. However, a study using a Canine Behavioural Assessment and Research Questionnaire (a validated questionnaire for assessing dogs’ behaviour) compared 30 breeds for aggressive behaviour and found that Chihuahuas were higher than average for human- and dog- directed aggression^48^. In addition to this, we hypothesize that bites by a small dog may be perceived as comical and thus more often uploaded online. It is also unclear if the breeds observed here are more likely to bite or to be more commonly owned^13,15,23^. In our study, male victims were over-represented. This trend has been noticed in previous publications^1,5,12,13,15^ but not to the same extent. It is therefore plausible that clips showing men are more often shared online. Our study included a similar proportion of adults to children and infants as those previously reported^12,16,17,49^, with children and infants being considerably more common victims than adults. Here, most bites were to the limbs, followed by bites to the face and neck area. Bites to face and neck area were more common among children and infants, which is also consistent with earlier reports^1,10,11,27^.

A variety of bite contexts were represented in our sample. Bites during play and benign interactions were particularly common, as reported before^10,14,16,18,24,50^. Play bites as well as behaviours that we labelled as benign interactions may be perceived as a normal part of human-dog behavioural repertoires and thus more frequently permitted and easier to film than other bite contexts. Moreover, bite victims may not see all bites as ‘true’ bites^35^, which could lead to bites in contexts such as play being more often uploaded online. In contrast to Reisner *et al*.^24^, we found that bites in the context of resources and resting were rare. The dissimilarity could arise due to differences in studied population: our sample consisted of all age groups, whilst Reisner *et al*.^24^ included only children. Alternatively, it could be due to these contexts being unlikely to be filmed.

Here we followed the classification used by Reisner *et al*.^24^ to aide comparison and because it permits categorisation based on the observed behaviours that can easily be recognised by an untrained observer. This terminology can, however, be misleading. For instance, previous research suggests that ‘benign’ interactions, as perceived by victims, may not be pleasant for dogs. Dogs may dislike being petted on top of their heads^49,51^, although we did not see a clear increase in tactile contact with head and neck areas before the bite.

Displacement and appeasement behaviours as well as postural changes and vocalisations were included in this analysis as they are often discussed as preceding a bite and taught as a part of bite-prevention education^38^. Closer to the time of the bite, dogs were more often coded as holding their body low or in an awkward position and their ears were more often observed to be in a non-neutral position. The postural changes have been linked with dogs experiencing acute distress in response to a fear-inducing stimuli^42^ and changes in ear carriage have been observed during training that involved painful stimuli^52^. It is plausible that these changes were detected here as some interactions leading to a bite may be painful or cause a distress to a dog. However, not all dogs in the videos show these changes and we also did not observe any clear changes in tail carriage pattern. Nonetheless, as postural changes may be easier to spot than some of the more subtle distance increasing behaviours, and as an increase in these behaviours was observed from approximately 30 seconds before the bite, bite-prevention messages should emphasise them more.

Following from the ladder of aggression theory^38^, behaviours such as lip licking, head turning are expected to escalate and be replaced with behaviours like snapping or growling in time before the bite. Head turning and full body turning as well as staring, stiffening, snapping, growling and frowning were observed proportionally more often in a build up to a bite, with head turning and staring dropping immediately before the bite, as would be expected from the ladder of aggression theory. We observed an increase in these behaviours approximately 20 seconds before the bite, which suggests that a person interacting with a dog does have time to alter their behaviour in response to these signs. However, as the increase in these behaviours is gradual, a person may not recognise their presence until later, if at all. A person may also recognise these signs and carry on interactions, assuming that a bite “would not happen to them”^26^. Other behaviours included in the ladder of aggression (like lip licking, paw lifting and sniffing) did not follow a clear pattern and sniffing and paw lifts were rarely observed. However, these behaviours may have escalated over a longer period of time, for instance a dog may have shown some of the behaviours from the lower steps of the ladder during previous interactions with a person, which would not have been captured in the video studied. Alternatively, these behaviours may not fit the pattern of behaviour progression proposed by the ladder of aggression theory. Overall, the postural changes were observed more often than other behaviours included in the ladder of aggression. Previous studies linked some of these behaviours (lip licking, paw lifts, head turns and yawning) with acute stress and pain^42,53^, emotional conflict^52^ and as a response to human facial expressions linked with a negative emotional valence^54^ which may be specific to some, but not all contexts in which bites occur.

Standing over a dog, petting and restraining a dog were seen proportionally more frequently closer to the bite, increasing approximately 20–30 seconds before. Other behaviours that did not result in contact and other tactile behaviours did not follow any clear pattern. The high prevalence of ‘standing over’ codes in the time preceding a bite suggests this particular behaviour should be emphasised in bite prevention training. The high frequency of petting and restraining behaviours makes prevention advice challenging, as these types of contacts are likely to occur when a person is familiar with the dog and interacts with a dog on daily basis, in a routine, habitual fashion. This results shows that dog owner education should emphasise the idea of all interactions with a dog, and in particular tactile interactions like petting, should be mutually consensual, i.e. only initiated by a person after a dog has already made a contact or otherwise shown an interest in being petted. In addition, restraining a dog e.g. in order to medicate it or prevent it from escaping may be hard to avoid and therefore requires additional care. It indicates the importance of teaching low-stress handling methods^55^.

The regression model with mean and 95% HDI estimates identified no significant differences in bite severity between bite contexts. This could be because there may be more similarities between bite contexts than differences, making the distinction between contexts difficult. However, the analysis of sample mean and bootstrap 95% CI suggested that territorial bites and bites in public spaces were more severe than other bites. Bites in the context of benign and unpleasant interactions and resting were less severe, which reflects previous research^27^. The bootstrap analysis also indicated that when a dog initiated the interactions (vs. a person), bite severity was greater. Bites in the context of benign interactions and unpleasant interactions may be more inhibited as the victim involved is likely to be more familiar with the dog. It is also plausible that the dog-initiated interactions in general may include more offensive aggression, whereas the human-initiated interactions may reflect more of the defensive aggression^56^. Different motivation to aggress could explain differences in severity as, in cases of defensive aggression, a dog may strive to warn off, which may result in a lesser severity of bite.

Overall, other predictor variables – and, in particular, the location of the bite on the body, age of the victim and size of the dog – were better at predicting the severity of a bite than context alone, although even here, the regression model with mean and 95% HDI estimates were not always significant. Again, this could be due to a small sample size or the way the severity measure was derived. In general, there may be numerous interactive effects between predictor variables that were not possible to explore in this study due to limited sample size and differences in number of videos in each context. The analysis showed that severity of a bite was correlated with the duration of a video, regardless of the context of interactions or other predictor variables. This could be because a person who was attacked for longer received more serious injuries or because longer attacks simply score higher severity mark. Moreover, not all variables which are often cited in literature as risk factors for bites were used as predictor variables in our model as it was not always possible to discern them from the video. We also did not include a breed as a predictor variable due to documented problems in recognising a breed based on visual characteristics^30^ and a small number of dogs in each breed category. One approach that could be utilised in the future on larger samples is systems-style modelling, such as using network analysis, that would identify interactive effects between variables that result in bites occurring and/or influence bite severity.

Using YouTube to study dog bites enabled us to carry out observations of bites of diverse severity, in naturalistic settings and across a range of contexts. The benefit of this approach is that permits studying human and dog behaviours preceding bites, which is not possible with other retrospective methods. However, the sample generated through YouTube search is subject to some biases as the frequency of bites in a given context and the victim and dog characteristics could reflect the likelihood with which these interactions are filmed and the self-selection bias for uploading videos online. The quality of videos and editing styles varied across the sample which meant that we could not collect a fine level of detail from each video. Small sample size meant that the analysis of body language had to be restricted to simple descriptive statistics, which is a further limitation of this method.

Moreover, our analysis is limited because the bite severity score reflects the perception of severity as observed in the video, as it is impossible to assess the full extent of each injury. It is plausible that as puncture wounds may be more difficult to identify in some videos, we sometimes under-estimated the severity. The same score on severity could, for example, reflect different elements of the bite; videos in the context of play may have a high severity score due to number of bites in a video whereas bites in the context of territorial aggression could have a high score due to puncture wounds and tearing movement of the dog, whereas in the reality, the later would cause more damage.

## Conclusion

In summary, this study used a novel approach to analyse human-dog interactions in naturalistic contexts. We found that despite potential biases of this sample, the demographic characteristics of the victims and dogs seen in YouTube bite videos reflect those found in previous publications. Although our analysis did not allow exploration of the causal relationship between human behaviour and dog bites, we observed that tactile contact with a dog increases approximately 20 seconds before a bite as does standing or leaning over a dog. Prevention messages could emphasise the risk of leaning over a dog and simply advise avoiding contact with a dog when possible or in doubt (for instance, when interacting with an unfamiliar dog). In a lead up to a bite, changes in dog body posture were more obvious than changes in appeasement and displacement behaviours. Some, but not all, appeasement and displacement behaviours described in the “ladder of aggression”^38^ were also observed.

## Electronic supplementary material

Supplementary Information File

Supplementary Dataset 1

Supplementary Dataset 2
