# Supplementary material for: Online videos indicate human and dog behaviour preceding dog bites and the context in which bites occur
Source: Sci Rep. 2018 May 8;8:7147. doi: 10.1038/s41598-018-25671-7 (PMC5940802; doi:10.1038/s41598-018-25671-7)

Supplementary Information File.

**Online videos indicate human and dog behaviour preceding dog bites and the context in which bites occur.**

Sara C. Owczarczak-Garstecka, Francine Watkins, Rob Christley, Carri Westgarth

**Supplement 1.** Dog behaviour ethogram

| **Group** | **Unit** | **Definition** |
| --- | --- | --- |
| Ears | neutral | Ears carried in a neutral position |
|  | not neutral | Ears pulled back towards the back of dog’s head, turned forwards or turned laterally backwards so that they come closer together ('bunny ears') |
| Tail | neutral | Tail is roughly in line with dog’s back, unless higher or lower cartridge is common for the dog’s/breed type |
|  | tucked | Tail is positioned between dog's hind legs, close to the belly, below the back line |
|  | high | Dog's tail is carried higher than normal for the breed/ type of dog, above the back line |
|  | wagging | Lateral or circular movement of the tail |
| Body posture | neutral | Head is carried not above/significantly below dog’s neck, legs are straight, body weight is evenly distributed on all legs of a dog |
|  | low | At least 2 legs are bent, dogs abdomen is lower to the ground than natural, head is carried low and below the neck line, body weight shifted backwards |
|  | high | Dog's head is up and above the neck line, body weight is shifted forwards |
|  | awkward | Dog is sitting or lying down in a tense or position, e.g. belly showing up, hips to one side, legs crossed |
| Displacement/ appeasement | Turn | The entire body or head rotates sideways in relation to the person or camera |
|  | Lick | Dog moves its tongue towards the nose or commissures and licks it |
|  | Panting | Mouth open, breathing rhythmically, tongue may or may not be protruding |
|  | Sniffing | Nose towards the ground/ person/ object/, breathing, movement of the nostril may be visible |
|  | Yawning | Mouth wide open and deep breath in |
|  | Paw lift | Dog lifts and bend front paw at an angle approximating 90 degrees (can be sitting or standing) |
|  | Shake off | Dog moves its head and body vigorously in opposite direction, giving an impression of shaking off water |
|  | Whale eye/ stare | The white of the eye is visible as e.g. the dog continues to look in one direction but its head is tilted/ The muscles around eyes are tense, the eyes are wide open, dog maintains a look for over 1 sec |
|  | Stiffening/freeze | Visible muscle tension around face, back or other areas that is maintained for over 1 second/dog is completely motionless for over 1 second |
|  | Frown | Dog wrinkles its nose and often lifts lips so that the teeth are visible |
|  | Snap | Dog opens and closes mouth rapidly near a person/ object as if to bite, but no contact is made |
| Movement | No movement | No locomotion visible |
|  | Slow | Dog is walking slowly |
|  | Medium | Dog is trotting/ walking quickly |
|  | Fast | Dog is running or moving quickly |
|  | Jumping | Dog is propelling itself upwards |
|  | Away | Dog is moving in a way that increases the distance from the person |
|  | Towards | Dogs is moving in a way that decreases the distance from the person |
|  | No change | Dog is moving but there are no changes in the distance from the person |
| Body position | Standing | Four paws are touching the ground simultaneously |
|  | Sitting | Dog’s hind legs are folded to the side or kicked to one side, rump is touching the floor and front legs are extended supporting the chest from making contact with the ground |
|  | Recumbent | Dog’s abdomen is touching the ground with its dorsal, caudal, or lateral side whilst legs are being extended forwards, curled close to the body or kicked to one side |
|  | Bow | Dog’s front legs are closer to the ground whilst its hind legs are straight as during play |
|  | Restrained | There is no change in distance because the dog is restrained (e.g. on the lead, cornered, hugged, grabbed by the collar etc.) |
| Contact | Contact- gentle | Dog is leaning into the person, resting its limbs or head on the person, licking, gently pawing the person or making a contact in other way that does not involve much pressure |
|  | Contact- intensive | Dog is jumping or pouncing at the person, running into a person, body barging, scratching, biting or making a contact in other way that does involve pressure. |
| Vocalisation | Growl | Dog makes a low guttural sound |
|  | Bark | Dog makes a short, loud vocalisation, often in a sequence. |
|  | Silent | Dog makes no vocalisations |
|  | Pain | Dog makes a short, high pitch vocalisation like a squeal or whimper |

**Supplement 2.** Human behaviour ethogram

| **Behaviour** | **Unit** | **Definition** |
| --- | --- | --- |
| Movement no contact | Head | Head tilt, head rotation or any other head movement |
|  | Body turn | A person rotates around their own axis |
|  | Slow arm | A person moves arm slowly near the dog |
|  | Rapid arm | A person moves arm quickly near the dog |
|  | Object | A person moves an object near the dog (e.g. a camera/ lead) |
|  | Slow leg | A person moves their leg slowly near the dog |
|  | Rapid leg | A person moves leg movement quickly near the dog |
|  | Stand over | A person is in a close proximity to a dog and shifts their body position so that their upper body is leaning over the dog |
| Movement | No movement | No locomotion visible |
|  | Slow | Walking or any other slow movement |
|  | Medium | Walking quickly or running slowly |
|  | Fast | Running quickly |
|  | Jumping | A person is propelling themselves upwards near a dog |
|  | Towards | A person is decreasing the distance from the dog |
|  | Away | A person is increasing the distance from the dog |
|  | No change | A person is moving, but the distance between them and the dog is not changing |
| Body Position | Sitting | Person’s buttocks are in contact with the chair or other surface; legs bear little or no weight of the person |
|  | Standing | All body weight relies on persons' legs |
|  | Crouching | A person is bend over, squatting or kneeling |
|  | Recumbent | A person is laying down |
| Contact | Petting | Gentle contact with the dog |
|  | Kissing | Person's lips touch the dog |
|  | Grooming | A person brushes the dog with a brush or towel |
|  | Hitting | Forceful contact between persons' hand and the dog |
|  | Restraining | A person holds dog’s collar or lead to prevent dog’s movement |
|  | Pushing | A person applies continues pressure to the dog to get increase the distance between themselves and the dog |
|  | Hitting with an object | A person hits the dog with an object or throws an object at the dog |
|  | Kicking | A forceful contact between person’s leg and the dog |
|  | Pulling towards | A person holds dog’s body part, collar or lead and moves the dog towards themselves |
|  | Pulling hair/skin/tail/ears | A person holds dog’s hair or skin and pulls |
|  | Holding a body part | A person encloses dog’s body part (paw, tail, and nose) in his or her hand. |
|  | Lifting | A person encircles the dog’s body and moves the dog up |
|  | Hugging | A person embraces the dog with two hands and applies gentle pressure |
| Vocalisation | Laughing | A person makes a laughing noise |
|  | Pain related vocalisation | A person cries/ squeals/ says ouch or swears in a way that suggests pain |
|  | Silent | A person is quiet |
|  | Normal talk | A person speaks normally |

**Supplement 3.** JAGS code

a) JAGS gamma with imputations

model {

for ( i in 1:N ) {

# likelihood; gamma parameterised with shape and rate

y[i] ~ dgamma( shape , rate[i] )

# linear predictor

rate[i] <- shape / exp(alpha + sum(Beta[1:P] * X[i,1:P]) + B_impute * I[i] )

# Log likelihood to calculate WAIC

logGamma[i] <- log(dgamma(y[i], shape, rate[i]))

}

shape ~ dunif( 0.001, 100) # prior on shape

alpha ~ dnorm( 0, (1/5^2) ) # prior on intercept (log scale)

for( p in 1:P ) { # priors on predictor coefficients (log-scale)

Beta[p] ~ dnorm( 0 , 1/1^2 ) # priors are regularising, pulled towards 0

}

B_impute ~ dnorm(0 , 1/1^2) # priors on the imputed variable’s coefficient, also

regularising

# missing data imputation

for(i in 1:N) {

X[i,impute_id] ~ dbern( theta ) # missing data is Bernoulli distributed

(0/1)

I[i] <- (X[i,impute_id] * 1) + ((1 - X[i,impute_id]) * -1) # convert 0/1 imputations to contrast

coding (-1/1)

} ### to feed back into the

regression

theta ~ dbeta(1,1) # prior distribution on probability of 0/1 value

}

b) JAGS hierarchical gamma no imputations

model {

for ( i in 1:N ) {

# likelihood; gamma parameterised with shape and rate

y[i] ~ dgamma( shape , rate[i] )

# linear predictor

rate[i] <- shape / exp(alpha + u[contexts[i]] + sum(Beta[1:P] * X[i,1:P]))

# Log likelihood to calculate WAIC

logGamma[i] <- log(dgamma(y[i], shape, rate[i]))

}

for( c in 1:Ncontexts ) {

u[c] ~ dnorm( 0 , 1/(sigma_contexts)^2 )

}

shape ~ dunif( 0.001, 100) # prior on shape

alpha ~ dnorm( 0, (1/5^2) ) # prior on intercept (log scale)

for( p in 1:P ){ # priors on predictor coefficients (log-scale)

Beta[p] ~ dnorm( 0 , 1/1^2 ) # priors are regularising, pulled towards 0

}

sigma_contexts ~ dunif(0.001, 100)

}

c) JAGS hierarchical gamma with imputations

model {

for ( i in 1:N ) {

# likelihood; gamma parameterised with shape and rate

y[i] ~ dgamma( shape , rate[i] )

# linear predictor

rate[i] <- shape / exp(alpha + u[contexts[i]] + sum(Beta[1:P] * X[i,1:P]) + B_impute * I[i] )

# Log likelihood to calculate WAIC

logGamma[i] <- log(dgamma(y[i], shape, rate[i]))

}

for( c in 1:Ncontexts ) {

u[c] ~ dnorm( 0 , 1/(sigma_contexts)^2 )

}

shape ~ dunif( 0.001, 100) # prior on shape

alpha ~ dnorm( 0, (1/5^2) ) # prior on intercept (log scale)

for( p in 1:P ){ # priors on predictor coefficients (log-scale)

Beta[p] ~ dnorm( 0 , 1/1^2 ) # priors are regularising, pulled towards 0

}

B_impute ~ dnorm(0 , 1/1^2) # priors on the imputed variable’s coefficient, also regularising

sigma_contexts ~ dunif(0.001, 100)

# missing data imputation

for(i in 1:N) {

X[i,impute_id] ~ dbern( theta ) # missing data is Bernoulli distributed

(0/1)

I[i] <- (X[i,impute_id] * 1) + ((1 - X[i,impute_id]) * -1) # convert 0/1 imputations to contrast

coding (-1/1)

} ### to feed back into the

regression

theta ~ dbeta(1,1) # prior distribution on probability of 0/1 value

}

**Supplement 4. R code**

#---------------------------------------------------------------------------

# Load relevant packages. If they are not loaded, run install.packages("package_name") first

#---------------------------------------------------------------------------

require(ggplot2)

require(rjags)

require(runjags)

require(rethinking)

require(ggplot2)

#============ convenience functions ================

# bootstrap confidence intervals

bootstrap_ci <- function(sample1 , sample2=NULL , n_samples=1e4,

ci_type = c("meanOnly","meanDiff"),

conf = c("lower","upper","both")) {

boots <- rep(list(list()), n_samples)

if( ci_type == "meanOnly") {

#if( dim(data)[2] > 1 ){ stop("Data should only have one column/be a vector") }

for(n in 1:n_samples) {

get_new_s1 <- sample(sample1, length(sample1), replace=TRUE)

boots[n] <- mean(get_new_s1)

}

}

if( ci_type == "meanDiff") {

for(n in 1:n_samples) {

get_new_s1 <- sample(sample1, length(sample1), replace=TRUE)

get_new_s2 <- sample(sample2, length(sample2), replace=TRUE)

boots[n] <- mean(get_new_s1) - mean(get_new_s2)

}

}

boots <- unlist(boots)

if( conf == "lower" ) {

res = quantile(as.numeric(boots), 0.025, na.rm = TRUE)[[1]]

}

if( conf == "upper" ) {

res = quantile(as.numeric(boots), 0.975,na.rm = TRUE)[[1]]

}

if( conf == "both" ) {

res = quantile(as.numeric(boots), c(0.025,0.975),na.rm = TRUE )[1:2]

}

res

}

# WAIC from log-likelihood

waic <- function( log_lik , data_length ) {

lppd <- sapply(1:data_length,

function(obs) log_sum_exp(log_lik[,obs]) - log(nrow(log_lik) )

)

p_waic <- sapply(1:data_length,

function(obs) var(log_lik[ , obs])

)

waic <- (-2) * ( sum(lppd) - sum(p_waic) )

se_waic <- sqrt( data_length * var( -2 * ( lppd - p_waic ) ) )

list(WAIC = waic ,

Standard.error = se_waic ,

In_sample_dev = (-2) * sum( lppd )

)

}

# convert WAIC into WAIC weights

WAICweights <- function( x ) {

best = min(x)

diffs = sapply(x, function(z) z - best )

num = sapply(diffs, function(z) exp( -(1/2) * z ) )

denom = sum(num)

weights = num / denom

round(weights,4)

}

#=========================================================================

#---------------------------------------------------------------------------

# Set working directory and load raw data

#---------------------------------------------------------------------------

setwd("~/Documents/PhD_NMBU/Saras_bootstrap/Final_analyses/Model_selection") # set your

working directory

my_data <- read.csv("raw_data.csv")

#---------------------------------------------------------------------------

# Variable names

#---------------------------------------------------------------------------

# dog_size = small, medium, large; location = inside/outside;

# C1 = resources; C2 = benign interaction; C3 = resting; C4 = painful;

# C5 = unpleasant; C6 = territorial; C7 = public spaces; C8 = play;

# initiated = human/dog; V1 = victim sex; V2 = victim age;

# skin puncture = no (1), yes (3); hold = no/yes; shake = no/yes;

#---------------------------------------------------------------------------

# Some data cleaning and calculating composite bite scores

#---------------------------------------------------------------------------

my_bite_vars <- c("num_bites","skin_puncture","hold","shake")

bite_vars <- my_data[,my_bite_vars]

my_data$composite <- ( bite_vars[,"num_bites"] * bite_vars[ , "skin_puncture"] ) +

bite_vars[,"hold"] + bite_vars[,"shake"]

#compositeW <- composite/my_data$duration

my_cat_vars <- c("dog_size", "victim_sex", "victim_age",

"bite_location", "location", "initiated")

my_data[ , my_cat_vars] <- apply(my_data[ , my_cat_vars] , 2 , as.factor )

my_data$durationZ <- as.numeric((my_data$duration -

mean(my_data$duration))/sd(my_data$duration))

#---------------------------------------------------------------------------

# Conduct model comparison on 15 competing models.

#---------------------------------------------------------------------------

# set JAGS things

n_chains <- 4; n_adapt <- 1000; n_burnin <- 10000; n_samples <- 50000; n_thin=10

#============ Model 1 ================

# ~ random contexts + duration + dog_size

options(na.action='na.pass') # keep NAs

X_mat <- model.matrix( ~ durationZ + dog_size,

data=my_data,

contrasts.arg = list(dog_size = "contr.sum"))[,-1]

jags_data <- list(N = nrow(my_data), X = X_mat,

P = ncol(X_mat)-1, y = my_data$composite ,

Ncontexts = length(unique(my_data$one_context)),

contexts = my_data$one_context )

fit_jags_1 <- run.jags(method = "parallel", model = "JAGS_hier_gamma_noImputation.txt", data =

jags_data,

monitor = c("alpha","Beta","shape", "sigma_contexts", "u", "logGamma"),

n.chains = n_chains, adapt = n_adapt,

burnin = n_burnin, sample = ceiling(n_samples/n_chains), thin = n_thin,

module = "glm"

)

coda_samples_1 <- as.mcmc.list(fit_jags_1)

mcmc_samples_1 <- as.matrix( coda_samples_1 )

waic_1 <- waic(log_lik = mcmc_samples_1[ , grep("logGamma",colnames(mcmc_samples_1))],

data_length = nrow(my_data))

#============ Model 2 ================

# ~ random contexts + duration + dog_size + victim_sex

options(na.action='na.pass') # keep NAs

X_mat <- model.matrix( ~ durationZ + dog_size + victim_sex,

data=my_data,

contrasts.arg = list(dog_size = "contr.sum",

victim_sex = "contr.sum"))[,-1]

jags_data <- list(N = nrow(my_data), X = X_mat,

P = ncol(X_mat)-1, y = my_data$composite ,

Ncontexts = length(unique(my_data$one_context)),

contexts = my_data$one_context )

fit_jags_2 <- run.jags(method = "parallel", model = "JAGS_hier_gamma_noImputation.txt", data =

jags_data,

monitor = c("alpha","Beta","shape", "sigma_contexts", "u", "logGamma"),

n.chains = n_chains, adapt = n_adapt,

burnin = n_burnin, sample = ceiling(n_samples/n_chains), thin = n_thin,

module = "glm"

)

coda_samples_2 <- as.mcmc.list(fit_jags_2)

mcmc_samples_2 <- as.matrix( coda_samples_2 )

waic_2 <- waic(log_lik = mcmc_samples_2[ , grep("logGamma",colnames(mcmc_samples_2))],

data_length = nrow(my_data))

#============ Model 3 ================

# ~ random contexts + duration + dog_size + victim_age

options(na.action='na.pass') # keep NAs

X_mat <- model.matrix( ~ durationZ + dog_size + victim_age,

data=my_data,

contrasts.arg = list(dog_size = "contr.sum",

victim_age = "contr.sum"))[,-1]

jags_data <- list(N = nrow(my_data), X = X_mat,

P = ncol(X_mat)-1, y = my_data$composite ,

Ncontexts = length(unique(my_data$one_context)),

contexts = my_data$one_context )

fit_jags_3 <- run.jags(method = "parallel", model = "JAGS_hier_gamma_noImputation.txt", data =

jags_data,

monitor = c("alpha","Beta","shape", "sigma_contexts", "u", "logGamma"),

n.chains = n_chains, adapt = n_adapt,

burnin = n_burnin, sample = ceiling(n_samples/n_chains), thin = n_thin,

module = "glm"

)

coda_samples_3 <- as.mcmc.list(fit_jags_3)

mcmc_samples_3 <- as.matrix( coda_samples_3 )

waic_3 <- waic(log_lik = mcmc_samples_3[ , grep("logGamma",colnames(mcmc_samples_3))],

data_length = nrow(my_data))

#============ Model 4 ================

# ~ random contexts + duration + dog_size + bite_location

options(na.action='na.pass') # keep NAs

X_mat <- model.matrix( ~ durationZ + dog_size + bite_location,

data=my_data,

contrasts.arg = list(dog_size = "contr.sum",

bite_location = "contr.sum"))[,-1]

jags_data <- list(N = nrow(my_data), X = X_mat,

P = ncol(X_mat)-1, y = my_data$composite ,

Ncontexts = length(unique(my_data$one_context)),

contexts = my_data$one_context )

fit_jags_4 <- run.jags(method = "parallel", model = "JAGS_hier_gamma_noImputation.txt", data =

jags_data,

monitor = c("alpha","Beta","shape", "sigma_contexts", "u", "logGamma"),

n.chains = n_chains, adapt = n_adapt,

burnin = n_burnin, sample = ceiling(n_samples/n_chains), thin = n_thin,

module = "glm"

)

coda_samples_4 <- as.mcmc.list(fit_jags_4)

mcmc_samples_4 <- as.matrix( coda_samples_4 )

waic_4 <- waic(log_lik = mcmc_samples_4[ , grep("logGamma",colnames(mcmc_samples_4))],

data_length = nrow(my_data))

#============ Model 5 (w/h imputation) ================

# ~ random contexts + duration + dog_size + initiated

options(na.action='na.pass') # keep NAs

X_mat <- model.matrix( ~ durationZ + dog_size + initiated,

data=my_data,

contrasts.arg = list(dog_size = "contr.sum",

initiated = "contr.sum"))[,-1]

jags_data <- list(N = nrow(my_data), X = X_mat, impute_id = ncol(X_mat),

P = ncol(X_mat)-1, y = my_data$composite ,

Ncontexts = length(unique(my_data$one_context)),

contexts = my_data$one_context )

fit_jags_5 <- run.jags(method = "parallel", model = "JAGS_hier_gamma_withImputation.txt", data =

jags_data,

monitor = c("alpha","Beta","Beta_impute", "theta", "shape", "sigma_contexts", "u", "I",

"logGamma"),

n.chains = n_chains, adapt = n_adapt,

burnin = n_burnin, sample = ceiling(n_samples/n_chains), thin = n_thin,

module = "glm"

)

coda_samples_5 <- as.mcmc.list(fit_jags_5)

mcmc_samples_5 <- as.matrix( coda_samples_5 )

waic_5 <- waic(log_lik = mcmc_samples_5[ , grep("logGamma",colnames(mcmc_samples_5))],

data_length = nrow(my_data))

#============ Model 6 ================

# ~ random contexts + duration + dog_size + victim_sex + victim_age

options(na.action='na.pass') # keep NAs

X_mat <- model.matrix( ~ durationZ + dog_size + victim_sex + victim_age,

data=my_data,

contrasts.arg = list(dog_size = "contr.sum",

victim_sex = "contr.sum",

victim_age = "contr.sum"))[,-1]

jags_data <- list(N = nrow(my_data), X = X_mat,

P = ncol(X_mat)-1, y = my_data$composite ,

Ncontexts = length(unique(my_data$one_context)),

contexts = my_data$one_context )

fit_jags_6 <- run.jags(method = "parallel", model = "JAGS_hier_gamma_noImputation.txt", data =

jags_data,

monitor = c("alpha","Beta","shape", "sigma_contexts", "u", "logGamma"),

n.chains = n_chains, adapt = n_adapt,

burnin = n_burnin, sample = ceiling(n_samples/n_chains), thin = n_thin,

module = "glm"

)

coda_samples_6 <- as.mcmc.list(fit_jags_6)

mcmc_samples_6 <- as.matrix( coda_samples_6 )

waic_6 <- waic(log_lik = mcmc_samples_6[ , grep("logGamma",colnames(mcmc_samples_6))],

data_length = nrow(my_data))

#============ Model 7 ================

# ~ random contexts + duration + dog_size + victim_sex + bite_location

options(na.action='na.pass') # keep NAs

X_mat <- model.matrix( ~ durationZ + dog_size + victim_sex + bite_location,

data=my_data,

contrasts.arg = list(dog_size = "contr.sum",

victim_sex = "contr.sum",

bite_location = "contr.sum"))[,-1]

jags_data <- list(N = nrow(my_data), X = X_mat,

P = ncol(X_mat)-1, y = my_data$composite ,

Ncontexts = length(unique(my_data$one_context)),

contexts = my_data$one_context )

fit_jags_7 <- run.jags(method = "parallel", model = "JAGS_hier_gamma_noImputation.txt", data =

jags_data,

monitor = c("alpha","Beta","shape", "sigma_contexts", "u", "logGamma"),

n.chains = n_chains, adapt = n_adapt,

burnin = n_burnin, sample = ceiling(n_samples/n_chains), thin = n_thin,

module = "glm"

)

coda_samples_7 <- as.mcmc.list(fit_jags_7)

mcmc_samples_7 <- as.matrix( coda_samples_7 )

waic_7 <- waic(log_lik = mcmc_samples_7[ , grep("logGamma",colnames(mcmc_samples_7))],

data_length = nrow(my_data))

#============ Model 8 (w/h imputation) ================

# ~ random contexts + duration + dog_size + victim_sex + initiated

options(na.action='na.pass') # keep NAs

X_mat <- model.matrix( ~ durationZ + dog_size + victim_sex + initiated,

data=my_data,

contrasts.arg = list(dog_size = "contr.sum",

victim_sex = "contr.sum" ,

initiated = "contr.sum"))[,-1]

jags_data <- list(N = nrow(my_data), X = X_mat, impute_id = ncol(X_mat),

P = ncol(X_mat)-1, y = my_data$composite ,

Ncontexts = length(unique(my_data$one_context)),

contexts = my_data$one_context )

fit_jags_8 <- run.jags(method = "parallel", model = "JAGS_hier_gamma_withImputation.txt", data =

jags_data,

monitor = c("alpha","Beta","B_impute", "theta", "shape", "sigma_contexts", "u", "I",

"logGamma"),

n.chains = n_chains, adapt = n_adapt,

burnin = n_burnin, sample = ceiling(n_samples/n_chains), thin = n_thin,

module = "glm"

)

coda_samples_8 <- as.mcmc.list(fit_jags_8)

mcmc_samples_8 <- as.matrix( coda_samples_8 )

waic_8 <- waic(log_lik = mcmc_samples_8[ , grep("logGamma",colnames(mcmc_samples_8))],

data_length = nrow(my_data))

#============ Model 9 ================

# ~ random contexts + duration + dog_size + victim_age + bite_location

options(na.action='na.pass') # keep NAs

X_mat <- model.matrix( ~ durationZ + dog_size + victim_age + bite_location,

data=my_data,

contrasts.arg = list(dog_size = "contr.sum",

victim_age = "contr.sum",

bite_location = "contr.sum"))[,-1]

jags_data <- list(N = nrow(my_data), X = X_mat,

P = ncol(X_mat)-1, y = my_data$composite ,

Ncontexts = length(unique(my_data$one_context)),

contexts = my_data$one_context )

fit_jags_9 <- run.jags(method = "parallel", model = "JAGS_hier_gamma_noImputation.txt", data =

jags_data,

monitor = c("alpha","Beta","shape", "sigma_contexts", "u", "logGamma"),

n.chains = n_chains, adapt = n_adapt,

burnin = n_burnin, sample = ceiling(n_samples/n_chains), thin = n_thin,

module = "glm"

)

coda_samples_9 <- as.mcmc.list(fit_jags_9)

mcmc_samples_9 <- as.matrix( coda_samples_9 )

waic_9 <- waic(log_lik = mcmc_samples_9[ , grep("logGamma",colnames(mcmc_samples_9))],

data_length = nrow(my_data))

#============ Model 10 (w/h imputation) ================

# ~ random contexts + duration + dog_size + victim_age + initiated

options(na.action='na.pass') # keep NAs

X_mat <- model.matrix( ~ durationZ + dog_size + victim_age + initiated,

data=my_data,

contrasts.arg = list(dog_size = "contr.sum",

victim_age = "contr.sum" ,

initiated = "contr.sum"))[,-1]

jags_data <- list(N = nrow(my_data), X = X_mat, impute_id = ncol(X_mat),

P = ncol(X_mat)-1, y = my_data$composite ,

Ncontexts = length(unique(my_data$one_context)),

contexts = my_data$one_context )

fit_jags_10 <- run.jags(method = "parallel", model = "JAGS_hier_gamma_withImputation.txt", data =

jags_data,

monitor = c("alpha","Beta","B_impute", "theta", "shape", "sigma_contexts", "u", "I",

"logGamma"),

n.chains = n_chains, adapt = n_adapt,

burnin = n_burnin, sample = ceiling(n_samples/n_chains), thin = n_thin,

module = "glm"

)

coda_samples_10 <- as.mcmc.list(fit_jags_10)

mcmc_samples_10 <- as.matrix( coda_samples_10 )

waic_10 <- waic(log_lik = mcmc_samples_10[ , grep("logGamma",colnames(mcmc_samples_10))],

data_length = nrow(my_data))

#============ Model 11 (w/h imputation) ================

# ~ random contexts + duration + dog_size + bite_location + initiated

options(na.action='na.pass') # keep NAs

X_mat <- model.matrix( ~ durationZ + dog_size + bite_location + initiated,

data=my_data,

contrasts.arg = list(dog_size = "contr.sum",

bite_location = "contr.sum" ,

initiated = "contr.sum"))[,-1]

jags_data <- list(N = nrow(my_data), X = X_mat, impute_id = ncol(X_mat),

P = ncol(X_mat)-1, y = my_data$composite ,

Ncontexts = length(unique(my_data$one_context)),

contexts = my_data$one_context )

fit_jags_11 <- run.jags(method = "parallel", model = "JAGS_hier_gamma_withImputation.txt", data =

jags_data,

monitor = c("alpha","Beta","B_impute", "theta", "shape", "sigma_contexts", "u", "I",

"logGamma"),

n.chains = n_chains, adapt = n_adapt,

burnin = n_burnin, sample = ceiling(n_samples/n_chains), thin = n_thin,

module = "glm"

)

coda_samples_11 <- as.mcmc.list(fit_jags_11)

mcmc_samples_11 <- as.matrix( coda_samples_11 )

waic_11 <- waic(log_lik = mcmc_samples_11[ , grep("logGamma",colnames(mcmc_samples_11))],

data_length = nrow(my_data))

#============ Model 12 ================

# ~ random contexts + duration + dog_size + victim_sex + victim_age + bite_location

options(na.action='na.pass') # keep NAs

X_mat <- model.matrix( ~ durationZ + dog_size + victim_sex + victim_age + bite_location,

data=my_data,

contrasts.arg = list(dog_size = "contr.sum",

victim_sex = "contr.sum", victim_age = "contr.sum",

bite_location = "contr.sum"))[,-1]

jags_data <- list(N = nrow(my_data), X = X_mat,

P = ncol(X_mat)-1, y = my_data$composite ,

Ncontexts = length(unique(my_data$one_context)),

contexts = my_data$one_context )

fit_jags_12 <- run.jags(method = "parallel", model = "JAGS_hier_gamma_noImputation.txt", data =

jags_data,

monitor = c("alpha","Beta","shape", "sigma_contexts", "u", "logGamma"),

n.chains = n_chains, adapt = n_adapt,

burnin = n_burnin, sample = ceiling(n_samples/n_chains), thin = n_thin,

module = "glm"

)

coda_samples_12 <- as.mcmc.list(fit_jags_12)

mcmc_samples_12 <- as.matrix( coda_samples_12 )

waic_12 <- waic(log_lik = mcmc_samples_12[ , grep("logGamma",colnames(mcmc_samples_12))],

data_length = nrow(my_data))

#============ Model 13 (w/h imputation) ================

# ~ random contexts + duration + dog_size + victim_sex + victim_age + initiated

options(na.action='na.pass') # keep NAs

X_mat <- model.matrix( ~ durationZ + dog_size + victim_sex + victim_age + initiated ,

data=my_data,

contrasts.arg = list(dog_size = "contr.sum",

victim_sex = "contr.sum" , victim_age = "contr.sum" ,

initiated = "contr.sum"))[,-1]

jags_data <- list(N = nrow(my_data), X = X_mat, impute_id = ncol(X_mat),

P = ncol(X_mat)-1, y = my_data$composite ,

Ncontexts = length(unique(my_data$one_context)),

contexts = my_data$one_context )

fit_jags_13 <- run.jags(method = "parallel", model = "JAGS_hier_gamma_withImputation.txt", data =

jags_data,

monitor = c("alpha","Beta","B_impute", "theta", "shape", "sigma_contexts", "u", "I",

"logGamma"),

n.chains = n_chains, adapt = n_adapt,

burnin = n_burnin, sample = ceiling(n_samples/n_chains), thin = n_thin,

module = "glm"

)

coda_samples_13 <- as.mcmc.list(fit_jags_13)

mcmc_samples_13 <- as.matrix( coda_samples_13 )

waic_13 <- waic(log_lik = mcmc_samples_13[ , grep("logGamma",colnames(mcmc_samples_13))],

data_length = nrow(my_data))

#============ Model 14 (w/h imputation) ================

# ~ random contexts + duration + dog_size + victim_sex + victim_age + bite_location + initiated

options(na.action='na.pass') # keep NAs

X_mat <- model.matrix( ~ durationZ + dog_size + victim_sex + victim_age + bite_location + initiated ,

data=my_data,

contrasts.arg = list(dog_size = "contr.sum" ,

victim_sex = "contr.sum", victim_age = "contr.sum",

bite_location = "contr.sum",

initiated = "contr.treatment"))[,-1]

jags_data <- list(N = nrow(my_data), X = X_mat,

P = ncol(X_mat)-1, impute_id = ncol(X_mat),

y = my_data$composite,

Ncontexts=length(unique(my_data$one_context)),

contexts = as.numeric(as.factor(my_data$one_context))

)

fit_jags_14 <- run.jags(method = "parallel", model = "JAGS_hier_gamma_withImputation.txt", data =

jags_data,

monitor = c("alpha","Beta","B_impute", "theta", "shape", "sigma_contexts", "u", "I",

"logGamma"),

n.chains = n_chains, adapt = n_adapt,

burnin = n_burnin, sample = ceiling(n_samples/n_chains), thin = n_thin,

module = "glm"

)

coda_samples_14 <- as.mcmc.list(fit_jags_14)

mcmc_samples_14 <- as.matrix( coda_samples_14 )

waic_14 <- waic(log_lik = mcmc_samples_14[ , grep("logGamma",colnames(mcmc_samples_14))],

data_length = nrow(my_data))

#============ Model 15 (w/h imputation) ================

# ~ fixed contexts + duration + dog_size + victim_sex + victim_age + bite_location + initiated

options(na.action='na.pass') # keep NAs

X_mat <- model.matrix( ~ one_context + durationZ + dog_size + victim_sex + victim_age +

bite_location + initiated ,

data=my_data,

contrasts.arg = list(one_context = "contr.sum", dog_size = "contr.sum" ,

victim_sex = "contr.sum", victim_age = "contr.sum",

bite_location = "contr.sum",

initiated = "contr.treatment"))[,-1]

jags_data <- list(N = nrow(my_data), X = X_mat,

P = ncol(X_mat)-1, impute_id = ncol(X_mat),

y = my_data$composite)

fit_jags_15 <- run.jags(method = "parallel", model = "JAGS_gamma_withImputation.txt", data =

jags_data,

monitor = c("alpha","Beta","B_impute", "theta", "shape", "I", "logGamma"),

n.chains = n_chains, adapt = n_adapt,

burnin = n_burnin, sample = ceiling(n_samples/n_chains), thin = n_thin,

module = "glm"

)

coda_samples_15 <- as.mcmc.list(fit_jags_15)

mcmc_samples_15 <- as.matrix( coda_samples_15 )

waic_15 <- waic(log_lik = mcmc_samples_15[ , grep("logGamma",colnames(mcmc_samples_15))],

data_length = nrow(my_data))

#---------------------------------------------------------------------------

# End of model selection: WAIC inspection and plotting

#---------------------------------------------------------------------------

# bundle WAIC estimates into a list

n_models = 15

waic_list <- list(waic_1 , waic_2, waic_3, waic_4, waic_5, waic_6, waic_7, waic_8, waic_9

, waic_10, waic_11, waic_12, waic_13, waic_14, waic_15)

waic_compare <- data.frame(model = paste("Model", 1:15, sep = " "),

WAIC = unlist(lapply(waic_list, function(z) z$WAIC)),

low_SE = unlist(lapply(waic_list, function(z) z$WAIC - z$Standard.error)),

high_SE = unlist(lapply(waic_list, function(z) z$WAIC + z$Standard.error)),

WAICweight = WAICweights(c(unlist(lapply(waic_list, function(z) z$WAIC))))

)

waic_compare$model <- factor(waic_compare$model, unique(waic_compare$model))

write.csv(waic_compare, "WAIC_results.csv", row.names = FALSE)

waic_compare <- read.csv("WAIC_results.csv")

waic_compare$model <- factor(waic_compare$model, unique(waic_compare$model))

ggplot(waic_compare, aes(x = model, y = WAIC) ) +

geom_errorbar(aes(ymin = low_SE, ymax = high_SE), lwd=0.6, width=0, col="#969696") +

geom_point(size=2) +

labs(x = "", y = "\nWAIC") +

theme_bw() +

theme(axis.text = element_text(colour="black", size=15),

axis.title = element_text(colour="black", size=20)) +

coord_flip()

ggsave("WAIC_plot.png", last_plot(), width=7, height = 4.5 )

#---------------------------------------------------------------------------

# Model 14 the best (albeit marginally), so save its posterior distribution

#---------------------------------------------------------------------------

capture.output(summary(fit_jags_14), file = "JAGS_output.csv")

write.csv(mcmc_samples_14, file = "mcmc_samples.csv", row.names = FALSE)

#---------------------------------------------------------------------------

# Post-processing of results

#---------------------------------------------------------------------------

mcmc_samples <- read.csv("mcmc_samples.csv")

#============ posterior predictive checks =============================

tiff(filename = "PPCheck.tiff", width = 1500, height = 1000, res=200)

par(mar=c(5,5,1,1))

hist(my_data$composite, col=col.alpha("darkblue", 0.7),

border="black",

prob=TRUE, xlim = c(range(my_data$composite)), ylim=c(0,0.25),

ylab = "Probability density", xlab = "Bite severity score",

main = "", breaks=20,

cex.lab = 2)

set.seed(12345)

pp_samples <- sample(1:nrow(mcmc_samples), 200, replace=FALSE)

for( pp in pp_samples ) {

shape <- mcmc_samples[ pp , "shape" ]

rate <- mcmc_samples[ pp , "shape" ] / exp(mcmc_samples[ pp , "alpha" ])

curve(dgamma(x, shape = shape, rate=rate), lwd=0.2, add = TRUE)

}

#curve(dgamma(x, shape = mean(shape), rate = mean(scale)), lwd=3, add = TRUE)

dev.off()

tiff(filename = "PPCheck_hists.tiff", width = 1800, height = 1000, res=200)

num_pp_checks <- 11

par(mfrow=c(3,4))

hist(my_data$composite, col=col.alpha("black",0.3), border="black",

xlim = c(range(my_data$composite)),

ylab = "", xlab = "",

main = "", breaks=20)

set.seed(12345)

pp_samples <- sample(1:nrow(mcmc_samples), num_pp_checks, replace=FALSE)

for( pp in pp_samples ) {

shape <- mcmc_samples[ pp , "shape" ]

scale <- mcmc_samples[ pp , "shape" ] / exp(mcmc_samples[ pp , "alpha" ])

hist(rgamma(n=143, shape = shape, rate = scale),

col=col.alpha("darkblue", 0.5), xlim=c(0,40),

ylab = "", xlab = "",

main = "", breaks=20)

}

dev.off()

#============ intra-class correlation =============================

ICC = mcmc_samples$sigma_contexts^2/(mcmc_samples$sigma_contexts^2

+log(1+1/mcmc_samples$shape) )

#============ plot duration by severity =============================

duration_comb <- my_data$durationZ #seq(min(my_data$durationZ), max(my_data$durationZ),

length=143)

pred <- sapply(duration_comb,

function(z) exp(mcmc_samples[,"alpha"] + mcmc_samples[,"Beta.1."]*z))

pred_mu <- apply(pred, 2, mean)

pred_hdi <- apply(pred, 2, function(z) HPDI( z, 0.95 ) )

duration_df <- data.frame(duration = my_data$duration,

duration_comb = duration_comb,

raw = my_data$composite,

mu = pred_mu,

hdi_low = pred_hdi[1,],

hdi_high = pred_hdi[2,]

)

ggplot(duration_df, aes(x = duration, y = raw)) +

geom_point(col = "black", alpha=0.5) +

geom_line(aes(x = duration, y = mu), lwd=1, col="darkblue", alpha=0.8) +

geom_line(aes(x = duration, y = hdi_low), linetype="dashed", lwd=1, col="darkblue", alpha=0.8) +

geom_line(aes(x = duration, y = hdi_high), linetype="dashed", lwd=1, col="darkblue", alpha=0.8) +

theme_bw() +

labs(x = "\nInteraction duration (s)",

y = "Bite severity score\n") +

theme(panel.grid = element_blank(),

axis.text = element_text(colour = "black", size=15),

axis.title = element_text(colour = "black", size=20))

ggsave(filename = "Duration_severity.png", last_plot(), width=6, height=5)

#============ plot bite contexts (random effects) =============================

re <- exp( mcmc_samples[,"alpha"] + mcmc_samples[,grep("u",colnames(mcmc_samples))][,-1])

re_plot <- data.frame(parameter = rep(c("Resources","Benign","Resting","Painful","Unpleasant",

"Territorial","Public","Play"),2),

type=rep(c("Mean & 95% HDI (model estimate)","Sample mean & bootstrap 95%

CI"),each=8),

mu = c(apply(re,2,mean),

aggregate(composite ~ one_context, data=my_data, mean )[,2]),

hdi_low = c(apply(re,2,function(z) HPDI(z,0.95))[1,],

aggregate(composite ~ one_context, data=my_data,

function(z)

bootstrap_ci(z, ci_type = "meanOnly",

conf="lower"))[,2]),

hdi_high = c(apply(re,2,function(z) HPDI(z,0.95))[2,],

aggregate(composite ~ one_context, data=my_data,

function(z)

bootstrap_ci(z, ci_type = "meanOnly",

conf="upper"))[,2]),

n = rep(as.vector( paste("n =",table(my_data$one_context) ) ),2)

)

#re_plot <- re_plot[with(re_plot,order(mu)),]

re_plot$parameter <- factor(re_plot$parameter, unique(re_plot$parameter) )

ggplot(re_plot, aes(x=parameter, y=mu, group=type, colour=type)) +

scale_colour_manual("", values = c("black", col.alpha("darkblue",0.5)) ) +

scale_y_continuous(limits=c(0,20), breaks=seq(0,20,5)) +

geom_hline(yintercept = exp(mean(mcmc_samples[,"alpha"])),

linetype="dashed", alpha=0.5) +

geom_point( position = position_dodge(0.7), size=2 ) +

geom_errorbar(aes(ymin=hdi_low, ymax=hdi_high, size=type), width=0,

position = position_dodge(0.7) ) +

scale_size_manual("", values=c(1,0.5), guide="none") +

annotate(geom = "text",

x = re_plot[re_plot$type=="Sample mean & bootstrap 95% CI", "parameter"],

y=re_plot[re_plot$type=="Sample mean & bootstrap 95% CI", "mu"],

label=re_plot[re_plot$type=="Sample mean & bootstrap 95% CI", "n"],

fontface="bold") +

labs(x = "", y = "\nEstimated bite severity score") +

theme_bw() +

theme(panel.grid = element_blank(),

axis.text.x = element_text(size=15, colour="black"),

axis.text.y = element_text(size=15, colour="black"),

axis.title.x = element_text(size=20, colour="black"),

legend.position = "top", legend.direction = "vertical",

legend.text = element_text(size=15)) +

coord_flip()

ggsave("Random_effect_contexts.png", last_plot(), width=7, height = 7)

#============ plot bite context contrasts against mean & bootrstapped CIs

=============================

context_contrasts <- data.frame(

benign.v.unpleasant = re[,"u.2."] - re[,"u.5."],

benign.v.territorial = re[,"u.2."] - re[,"u.6."],

benign.v.public = re[,"u.2."] - re[,"u.7."], benign.v.play = re[,"u.2."] - re[,"u.8."],

unpleasant.v.territorial = re[,"u.5."] - re[,"u.6."],

unpleasant.v.public = re[,"u.5."] - re[,"u.7."], unpleasant.v.play = re[,"u.5."] - re[,"u.8."],

territorial.v.public = re[,"u.6."] - re[,"u.7."],

territorial.v.play = re[,"u.6."] - re[,"u.8."],

public.v.play = re[,"u.7."] - re[,"u.8."] )

raw_re_mus <- c(

benign.v.unpleasant = mean(my_data[my_data$one_context=="C2", "composite"]) -

mean(my_data[my_data$one_context=="C5", "composite"]) ,

benign.v.territorial = mean(my_data[my_data$one_context=="C2", "composite"]) -

mean(my_data[my_data$one_context=="C6", "composite"]) ,

benign.v.public = mean(my_data[my_data$one_context=="C2", "composite"]) -

mean(my_data[my_data$one_context=="C7", "composite"]) ,

benign.v.play = mean(my_data[my_data$one_context=="C2", "composite"]) -

mean(my_data[my_data$one_context=="C8", "composite"]) ,

unpleasant.v.territorial = mean(my_data[my_data$one_context=="C5", "composite"]) -

mean(my_data[my_data$one_context=="C6", "composite"]) ,

unpleasant.v.public = mean(my_data[my_data$one_context=="C5", "composite"]) -

mean(my_data[my_data$one_context=="C7", "composite"]) ,

unpleasant.v.play = mean(my_data[my_data$one_context=="C5", "composite"]) -

mean(my_data[my_data$one_context=="C8", "composite"]) ,

territorial.v.public = mean(my_data[my_data$one_context=="C6", "composite"]) -

mean(my_data[my_data$one_context=="C7", "composite"]) ,

territorial.v.play = mean(my_data[my_data$one_context=="C6", "composite"]) -

mean(my_data[my_data$one_context=="C8", "composite"]) ,

public.v.play = mean(my_data[my_data$one_context=="C7", "composite"]) -

mean(my_data[my_data$one_context=="C8", "composite"])

)

raw_low_cis <- c(

benign.v.unpleasant = bootstrap_ci(my_data[my_data$one_context=="C2", "composite"],

my_data[my_data$one_context=="C5", "composite"],

ci_type="meanDiff", conf="lower") ,

benign.v.territorial = bootstrap_ci(my_data[my_data$one_context=="C2", "composite"],

my_data[my_data$one_context=="C6", "composite"],

ci_type="meanDiff", conf="lower") ,

benign.v.public = bootstrap_ci(my_data[my_data$one_context=="C2", "composite"],

my_data[my_data$one_context=="C7", "composite"],

ci_type="meanDiff", conf="lower") ,

benign.v.play = bootstrap_ci(my_data[my_data$one_context=="C2", "composite"],

my_data[my_data$one_context=="C8", "composite"],

ci_type="meanDiff", conf="lower") ,

unpleasant.v.territorial = bootstrap_ci(my_data[my_data$one_context=="C5", "composite"],

my_data[my_data$one_context=="C6", "composite"],

ci_type="meanDiff", conf="lower") ,

unpleasant.v.public = bootstrap_ci(my_data[my_data$one_context=="C5", "composite"],

my_data[my_data$one_context=="C7", "composite"],

ci_type="meanDiff", conf="lower") ,

unpleasant.v.play = bootstrap_ci(my_data[my_data$one_context=="C5", "composite"],

my_data[my_data$one_context=="C8", "composite"],

ci_type="meanDiff", conf="lower") ,

territorial.v.public = bootstrap_ci(my_data[my_data$one_context=="C6", "composite"],

my_data[my_data$one_context=="C7", "composite"],

ci_type="meanDiff", conf="lower") ,

territorial.v.play = bootstrap_ci(my_data[my_data$one_context=="C6", "composite"],

my_data[my_data$one_context=="C8", "composite"],

ci_type="meanDiff", conf="lower") ,

public.v.play = bootstrap_ci(my_data[my_data$one_context=="C7", "composite"],

my_data[my_data$one_context=="C8", "composite"],

ci_type="meanDiff", conf="lower")

)

raw_high_cis <- c(

benign.v.unpleasant = bootstrap_ci(my_data[my_data$one_context=="C2", "composite"],

my_data[my_data$one_context=="C5", "composite"],

ci_type="meanDiff", conf="upper") ,

benign.v.territorial = bootstrap_ci(my_data[my_data$one_context=="C2", "composite"],

my_data[my_data$one_context=="C6", "composite"],

ci_type="meanDiff", conf="upper") ,

benign.v.public = bootstrap_ci(my_data[my_data$one_context=="C2", "composite"],

my_data[my_data$one_context=="C7", "composite"],

ci_type="meanDiff", conf="upper") ,

benign.v.play = bootstrap_ci(my_data[my_data$one_context=="C2", "composite"],

my_data[my_data$one_context=="C8", "composite"],

ci_type="meanDiff", conf="upper") ,

unpleasant.v.territorial = bootstrap_ci(my_data[my_data$one_context=="C5", "composite"],

my_data[my_data$one_context=="C6", "composite"],

ci_type="meanDiff", conf="upper") ,

unpleasant.v.public = bootstrap_ci(my_data[my_data$one_context=="C5", "composite"],

my_data[my_data$one_context=="C7", "composite"],

ci_type="meanDiff", conf="upper") ,

unpleasant.v.play = bootstrap_ci(my_data[my_data$one_context=="C5", "composite"],

my_data[my_data$one_context=="C8", "composite"],

ci_type="meanDiff", conf="upper") ,

territorial.v.public = bootstrap_ci(my_data[my_data$one_context=="C6", "composite"],

my_data[my_data$one_context=="C7", "composite"],

ci_type="meanDiff", conf="upper") ,

territorial.v.play = bootstrap_ci(my_data[my_data$one_context=="C6", "composite"],

my_data[my_data$one_context=="C8", "composite"],

ci_type="meanDiff", conf="upper") ,

public.v.play = bootstrap_ci(my_data[my_data$one_context=="C7", "composite"],

my_data[my_data$one_context=="C8", "composite"],

ci_type="meanDiff", conf="upper")

)

re_contrasts <- data.frame(parameter = rep(colnames(context_contrasts),2),

type = rep(c("95% HDI includes zero (model estimate)",

"Sample mean & bootstrap 95% CI"),

each = length(colnames(context_contrasts))),

mu = c(apply(context_contrasts,2,mean), raw_re_mus),

hdi_low = c(apply(context_contrasts,2,function(z) HPDI(z,0.95))[1,],

raw_low_cis),

hdi_high = c(apply(context_contrasts,2,function(z) HPDI(z,0.95))[2,],

raw_high_cis )

)

re_contrasts$parameter <- factor(re_contrasts$parameter, unique(re_contrasts$parameter))

my_re_labels <- c("Benign - unpleasant", "Benign - territorial", "Benign - Public",

"Benign - play", "Unpleasant - territorial", "Unpleasant - public",

"Unpleasant - play", "Territorial - public", "Territorial - play",

"Public - play")

ggplot(re_contrasts, aes(x=parameter, y=mu, group=type, colour=type)) +

scale_x_discrete(labels=my_re_labels) +

scale_colour_manual("", values = c("darkgray", col.alpha("darkblue",0.5)) ) +

geom_hline(yintercept = 0, linetype="dashed",alpha=0.5) +

geom_point( position = position_dodge(0.7), size=2 ) +

geom_errorbar(aes(ymin=hdi_low, ymax=hdi_high, size=type), width=0,

position = position_dodge(0.7) ) +

scale_size_manual("", values=c(1,0.5), guide="none") +

labs(x = "", y = "\nEstimated differences in bite severity") +

theme_bw() +

theme(panel.grid = element_blank(),

axis.text.x = element_text(size=15, colour="black"),

axis.text.y = element_text(size=15, colour="black"),

axis.title.x = element_text(size=20, colour="black"),

legend.position = "top", legend.direction = "vertical",

legend.text = element_text(size=15)) +

coord_flip()

ggsave("Re_contrast_plot.png", last_plot(), width=8, height=8)

#============ plot fixed effect results against sample mean and 95% bootstrap CIs

=============================

fe_preds <- with(mcmc_samples,

data.frame(

small = alpha + Beta.2.,

med = alpha + Beta.3.,

large = alpha + (Beta.2.+ Beta.3.)*-1 ,

male = alpha + Beta.4.,

female = alpha + Beta.4.*-1,

infant = alpha + Beta.5.,

child = alpha + Beta.6.,

adult = alpha + (Beta.5.+Beta.6.)*-1,

face = alpha + Beta.7. ,

limbs = alpha + Beta.8. ,

multiple = alpha + Beta.9. ,

other = alpha + (Beta.7.+Beta.8.+Beta.9.)*-1,

dog_initiates = alpha + B_impute ,

human_initiates = alpha + B_impute*-1

)

)

fe_preds_orig <- as.data.frame( apply(fe_preds, 2 , exp) )

fe_contrasts <- with(fe_preds_orig,

data.frame(

small.v.medium = small - med,

small.v.large = small - large,

medium.v.large = med - large,

female.v.male = female- male,

infant.v.child = infant - child, infant.v.adult = infant - adult, child.v.adult = child - adult,

dog.v.human_initiates = dog_initiates - human_initiates,

face.v.limbs = face - limbs, face.v.multiple = face - multiple,

limbs.v.multiple = limbs - multiple

))

fe_plot <- data.frame(parameter = colnames(fe_contrasts),

mu = apply(fe_contrasts,2,mean),

hdi_low = apply(fe_contrasts,2,function(z) HPDI(z,0.95))[1,],

hdi_high = apply(fe_contrasts,2,function(z) HPDI(z,0.95))[2,])

fe_plot$decision <- ifelse(fe_plot$hdi_low > 0 | fe_plot$hdi_high < 0 ,

"95% HDI excludes zero",

"95% HDI includes zero")

fe_plot$parameter <- factor(fe_plot$parameter, unique(fe_plot$parameter))

my_labels <- c("Dog size: small - medium", "Dog size: small - large", "Dog size: medium - large",

"Victim sex: female - male", "Victim age: infant - child",

"Victim age: infant - adult","Victim age: child - adult",

"Initiated: dog - human", "Location: face - limbs",

"Location: face - multiple", "Location: limbs - multiple")

ggplot(fe_plot, aes(x=parameter, y=mu,colour=decision)) +

scale_x_discrete(labels = my_labels) +

scale_colour_manual("", values = c("black","darkgrey")) +

geom_hline(yintercept = 0, linetype="dashed",alpha=0.5) +

geom_point( position = position_dodge(0.7), size=2 ) +

geom_errorbar(aes(ymin=hdi_low, ymax=hdi_high), width=0, size=1,

position = position_dodge(0.7) ) +

labs(x = "", y = "\nEstimated differences in bite severity") +

labs(x = "", y = "\nEstimated differences in bite severity") +

theme_bw() +

theme(panel.grid = element_blank(),

axis.text.x = element_text(size=15, colour="black"),

axis.text.y = element_text(size=15, colour="black"),

axis.title.x = element_text(size=20, colour="black"),

legend.position = "top", legend.direction = "vertical",

legend.text = element_text(size=15)) +

coord_flip()

ggsave("fixedEffect_plot.png", last_plot(), width=8, height = 8)

#=======Make nice summary tables===========================

re_table <- data.frame(Variable = c("Resources","Benign","Resting","Painful","Unpleasant",

"Territorial","Public","Play","Standard deviation"),

mu = round(c ( apply(re, 2, mean ),

mean(mcmc_samples[,"sigma_contexts"])),2),

hdi_low = round(c(apply(re, 2, function(z) HPDI(z, 0.95))[1,],

HPDI(mcmc_samples[,"sigma_contexts"], 0.95)[1]),2),

hdi_high = round(c(apply(re, 2, function(z) HPDI(z, 0.95))[2,],

HPDI(mcmc_samples[,"sigma_contexts"], 0.95)[2]),2) )

rownames(re_table) <- 1:nrow(re_table)

write.csv(re_table, "re_table.csv", row.names = FALSE)

contrasts_table <- data.frame(Variable = c(rep("contexts", 10) ,

rep("dog_size", 3),

"sex", rep("age", 3),

"initiates",

rep("bite_location", 3) ),

Contrast = c(as.character(re_contrasts$parameter[1:10] ),

colnames(fe_contrasts)) ,

mu = round(c(re_contrasts$mu[1:10],

apply(fe_contrasts, 2, mean )),2),

hdi_low = round(c(re_contrasts$hdi_low[1:10],

apply(fe_contrasts, 2, function(z) HPDI(z,0.95) )[1,]),2),

hdi_high = round(c(re_contrasts$hdi_high[1:10],

apply(fe_contrasts, 2, function(z) HPDI(z,0.95) )[2,]),2)

)

rownames(contrasts_table) <- 1:nrow(contrasts_table)

write.csv(contrasts_table, "contrasts_table.csv", row.names = FALSE)

**Supplement 5.** WAIC model comparison

| model | WAIC | low_SE | high_SE | WAICweight |
| --- | --- | --- | --- | --- |
| Model 1 | 775.996 | 750.3854 | 801.6065 | 0.0082 |
| Model 2 | 778.4435 | 752.6112 | 804.2759 | 0.0024 |
| Model 3 | 780.9484 | 755.0218 | 806.8751 | 7.00E-04 |
| Model 4 | 782.7069 | 756.8942 | 808.5196 | 3.00E-04 |
| Model 5 | 781.3688 | 754.726 | 808.0115 | 6.00E-04 |
| Model 6 | 782.9422 | 756.9871 | 808.8974 | 3.00E-04 |
| Model 7 | 784.701 | 758.8243 | 810.5778 | 1.00E-04 |
| Model 8 | 783.4538 | 756.7173 | 810.1904 | 2.00E-04 |
| Model 9 | 783.5339 | 757.817 | 809.2507 | 2.00E-04 |
| Model 10 | 782.4248 | 755.7366 | 809.113 | 3.00E-04 |
| Model 11 | 768.5854 | 742.9576 | 794.2132 | 0.3326 |
| Model 12 | 785.5065 | 759.8029 | 811.2101 | 1.00E-04 |
| Model 13 | 784.539 | 757.8237 | 811.2543 | 1.00E-04 |
| Model 14 | 767.9832 | 742.2146 | 793.7518 | 0.4495 |
| Model 15 | 769.5578 | 743.9294 | 795.1862 | 0.2045 |

**Supplement 5.** Diagram comparing different models against WAIC.


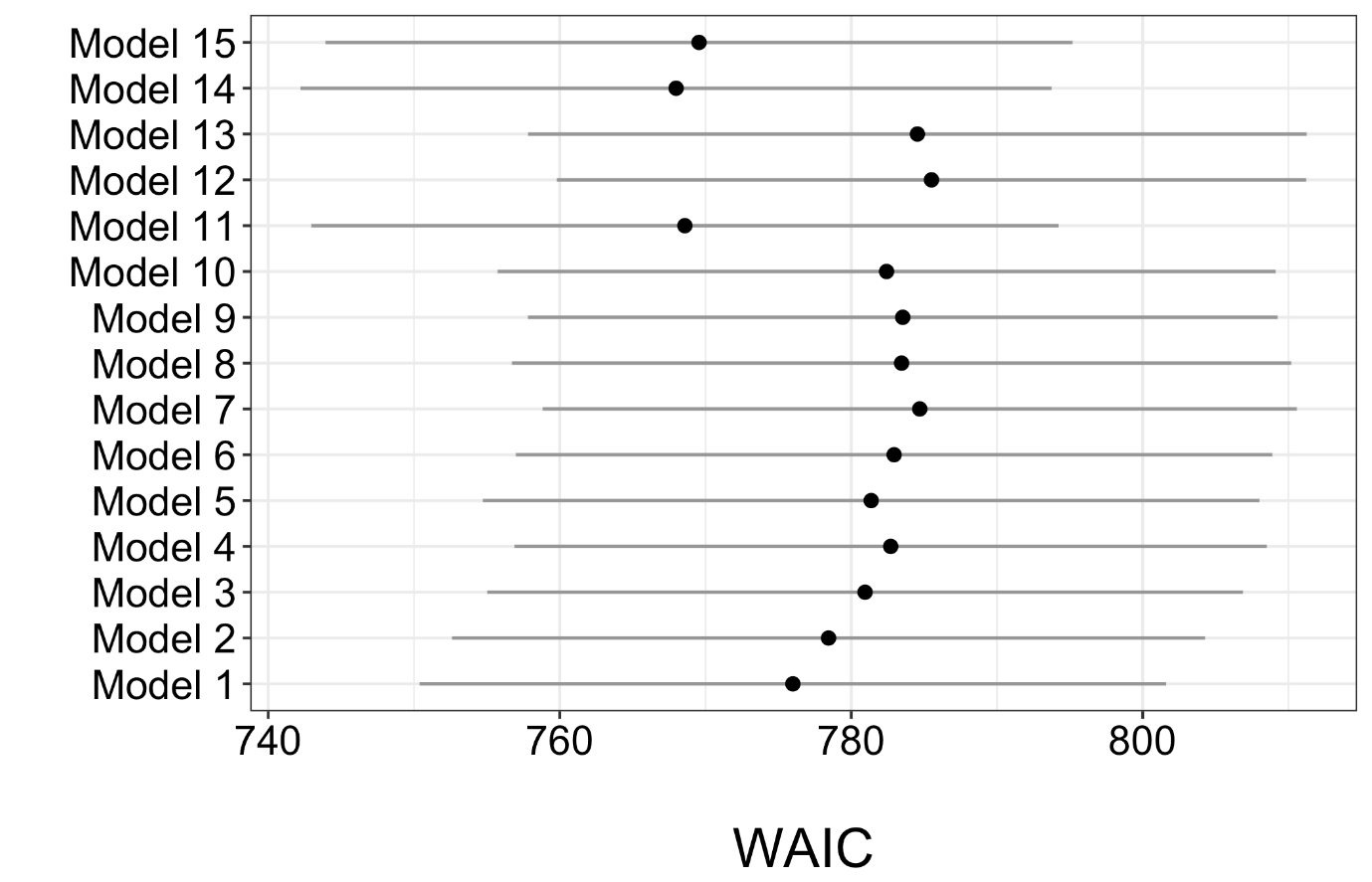


**Supplement 6.** Contrast table comparing estimated bite severity between contexts and different

demographic variables.

| Variable | Contrast | mu | hdi_low | hdi_high |
| --- | --- | --- | --- | --- |
| contexts | benign.v.unpleasant | 0.39 | -1.09 | 2.07 |
| contexts | benign.v.territorial | -0.74 | -3.24 | 1.1 |
| contexts | benign.v.public | -0.94 | -3.47 | 0.97 |
| contexts | benign.v.play | -0.75 | -2.73 | 0.84 |
| contexts | unpleasant.v.territorial | -1.13 | -3.89 | 0.85 |
| contexts | unpleasant.v.public | -1.33 | -4.2 | 0.71 |
| contexts | unpleasant.v.play | -1.13 | -3.41 | 0.59 |
| contexts | territorial.v.public | -0.2 | -2.41 | 1.97 |
| contexts | territorial.v.play | -0.01 | -2.03 | 2.27 |
| contexts | public.v.play | 0.19 | -1.83 | 2.54 |
| dog_size | small.v.medium | -1.08 | -3.06 | 0.79 |
| dog_size | small.v.large | -0.71 | -2.37 | 1.01 |
| dog_size | medium.v.large | 0.38 | -1.32 | 2.11 |
| sex | female.v.male | -0.38 | -1.9 | 1.11 |
| age | infant.v.child | 2.17 | 0.14 | 4.37 |
| age | infant.v.adult | 0.56 | -1.59 | 2.75 |
| age | child.v.adult | -1.61 | -3.16 | -0.08 |
| initiates | dog.v.human_initiates | 1.1 | -0.51 | 2.72 |
| bite_location | face.v.limbs | -0.95 | -2.85 | 1.06 |
| bite_location | face.v.multiple | -7.76 | -13.37 | -2.62 |
| bite_location | limbs.v.multiple | -6.82 | -12.15 | -2.31 |

**Supplement 7.** The effect of interaction duration on bite severity (mean= solid line, dotted lines =

95% HDI).


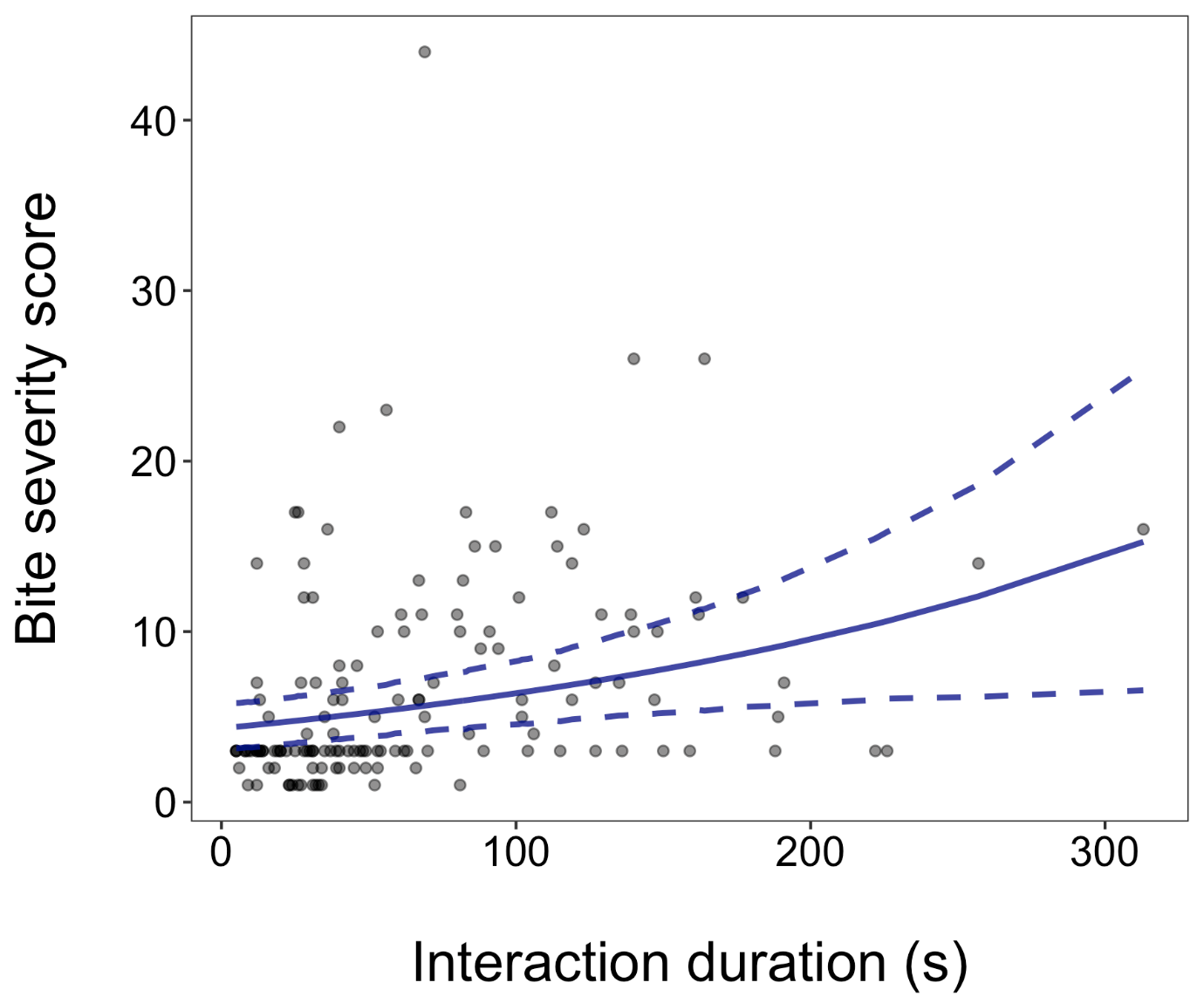

Supplement: Supplementary file 1 — Supplementary Information File [file 41598_2018_25671_MOESM1_ESM.docx]
